# Bayesian optimization and machine learning for vaccine formulation development

**DOI:** 10.1371/journal.pone.0324205

**Published:** 2025-06-11

**Authors:** Lillian Li, Sung-In Back, Jian Ma, Yawen Guo, Thomas Galeandro-Diamant, Didier Clénet

**Affiliations:** 1 Vaccine CMC Development & Supply, Sanofi, Toronto, Ontario, Canada; 2 Vaccine CMC Development & Supply, Sanofi, Marcy-L’ Etoile, France; 3 ChemAI Ltd. West Hill House, Allerton Hill, Chapel Allerton, Leeds, United Kingdom; Icahn School of Medicine at Mount Sinai Department of Pharmacological Sciences, UNITED STATES OF AMERICA

## Abstract

Developing vaccines with a better stability is an area of improvement to meet the global health needs of preventing infectious diseases. With the advancement of data science and artificial intelligence, innovative approaches have emerged. This manuscript highlights the applications of machine learning through two cases in which Bayesian optimization was used to develop viral vaccine formulations. The two case studies monitored the critical quality attributes of virus A in liquid form by infectious titer loss and virus B in freeze-dried form by glass transition temperature. Stepwise analysis and model optimization demonstrated progressive improvements of model quality and prediction accuracy. The cross-validation matrices of the models’ predictions showed high R² and low root mean square errors, indicating their reliability. The prediction accuracy of models was further validated by using test datasets. Model analysis using prediction error plot, Shapeley Additive exPlanations, permutation importance, etc. can provide additional insights into relations between model and experimental design, the influence of features of interest, and non-linear responses. Overall, Bayesian optimization is a useful complementary tool in formulation development that can help scientists make effective data-driven decisions.

## Introduction

Vaccine is an important type of pharmaceutical product that provides a simple and economic way of preventing infectious diseases, while saving millions of lives from pandemics worldwide [1]. However, developing a new vaccine raises a host of challenges, from designing and stabilizing a new antigen up to distributing the vaccine doses to target populations. After a vaccine product is shipped out of the manufacturing site, the challenge still lies in ensuring its critical quality attributes (CQAs), stability, and effectiveness throughout its shelf-life. Hence, huge efforts are made to enhance product thermal stability, minimize distribution cost, and avoid the complexities of global cold-chain management [2–4]. The current World Health Organization (WHO) recommendations [5] and International Conference on Harmonisation (ICH) [6] provide guidance for defining a product shelf-life. With a good understanding of the product CQAs, empirical approach, high throughput excipients screening, and design of experiments (DoE) are often deployed to optimize the formulation of a new vaccine product. In combination with advanced kinetic modeling (AKM), appropriate predictions of product stability can be done both at recommended storage conditions (i.e., 5°C) and during unexpected temperature excursions (cold-chain breaks) [7–10]. This can be laborious and often requires a tremendous amount of resources to meet global project objectives under competitive project timelines. First, the empirical “trial-and-error” or DoE can only assess a limited number of necessary variables during pre-formulation and formulation development [11]. Second, during the development of biopharmaceuticals, such as vaccines, impact on the critical parameters can be carried over from upstream bioprocessing. As a result, the design space covered by DoE approach is unable to capture responses with limited number of variables beyond a single unit operation. This limitation does not allow to systematically study critical parameters, i.e., the interactions between formulation variables and process variables, during a product formulation development [12].

With the recent advances in the field of computer science, artificial intelligence (AI) and machine learning (ML) have become powerful resources, enabling the use of data-driven predictive modeling tools for in silico vaccine development [13]. Among them, the Bayesian optimization (BO) algorithm is an iterative process in which unknown system responses are modelled using Gaussian process (GP) models. The GP models are then used to compute an acquisition function that assesses experimental design with regard to the set objectives [14]. Subsequently, an experiment with optimized features of interest (FOI) is suggested either in an unexplored area of the design space or in an area where the ML model predicts an optimal result. The suggested experiment is then performed and the new data point is added to the dataset [14]. This cycle of generating new experimental data and feeding these additional experimental data to the database can be repeated. With each iteration, the model quality is progressively improved. For high-dimensional optimization problems, this approach has been shown to outperform and be less labor-intensive than “trial and error” and DoE approaches, achieving significant popularity in chemistry [15,16]. Thus, leveraging ML, can help achieve effective experimental design [16], protein engineering, developability assessment, and formulation [17], with capacities that surpass traditional processes and methods in biopharmaceutical development. In recent years, the trend of exponential growth for ML-based applications has also been seen in the pharma industry. ML applications have been used at all stages of drug discovery, from the identification of novel drug targets to the optimization of clinical trials’ design [18]. Some reported use cases include using ML for screening antigen candidates for infectious diseases such as COVID-19 [13], and optimization of downstream process for production of monoclonal antibodies [19]. Overall, ML is a powerful tool that enables scientists to find valuable information through data mining and to predict experimental outcomes based on sets of parameter values [20], even using data from failed experiments [21]. However, as of now, there are very few reports documenting the use of a ML-based application to help identify potential stabilizers and excipients for specific biological formulations [16,22].

To overcome the limitations of conventional vaccine excipient screening, such as it being empirical, resource intensive, and time-consuming, and limited inputs and outputs, a proof of concept (POC) study using BO and ML-based modeling was conducted. In this paper, we present the POC through two vaccine formulation development case studies. In case study 1, we outline how to leverage the BO based modeling, methods to analyze the experimental data, and ultimately, how to utilize model prediction for formulation improvements. One of the excipients, recombinant Human Serum Albumin (rHSA), a soluble protein that is often used as a supplement in upstream bioprocessing and/or as an excipient, was further analyzed through ML to gain a deeper understanding of its role and to identify its optimal concentration for the stability of a live-attenuated virus A vaccine formulation. The stabilizing effect of rHSA predicted by the model was found to be comparable to that of many other live-attenuated virus vaccines in the literature [23–27]. The same ML approach was applied to identify stabilizing excipients in live-attenuated virus B vaccine formulations. The prediction accuracy of glass transition temperature (Tg’) values are presented for case study 2. The predicted values generated by the ML-based model were close to experimental values and within a small error margin. Together, the two case studies demonstrate that the BO-based modeling is a viable tool for vaccine formulation development, capable of accelerating projects even with small datasets and limited experimental budget. It is also suitable to analyze different types of formulations and characteristics.

## Materials and methods

### Vaccine candidates

The potential of BO-based modeling for formulation development was investigated with two types of vaccine candidates. The excipients screened for both case studies are commonly-used compounds such as amino acids, antioxidants and chelating agents, sugars and polyols, mono- and bi-valent salts, polymers and proteins, surfactants, and buffer agents [11].

Case study 1 involved developing and optimizing potential vaccine formulations for a genetically modified live-attenuated virus A. Infectious titer was used as the main stability indicating assay, as it directly correlates to viral activity and the potency of the vaccine candidate. Thus, a significant decrease in infectious titer reflects a drop in viral activity. All samples were tested in duplicates in each experiment. The formulation screening measured titer loss at 37°C after one-week of incubation [5]. The other key attributes were pH and physical appearance. In this case, a neutral pH at fixed value was used for all of experiments. Both pH and physical appearance remained unchanged throughout the study.

Case study 2 examined another live-attenuated virus B, developed as a vaccine in freeze-dried (FD) process. It focused on assessing which excipient or excipients combinations could help optimize the Tg’ of the concentrated bulk solution during the FD process of the vaccine candidate. The Tg’ during freezing is the temperature at which a product undergoes a glass transition from a viscous state (due to the crystallization of ice) to a glassy state, becoming more rigid and blocking molecular cooperative motions. The rationale for optimizing this parameter was that as ice crystals form and grow, they can damage the product’s structure and affect its stability. Thus, a higher Tg’ facilitates the formation of the glassy state, reducing the possibility of physical and chemical changes during the FD process, helping to prevent the collapse phenomenon, and improving the overall efficiency of FD [28].

In practice, a Tg’ above -36°C is expected during development of vaccine formulation to achieve a one-day industrial cycle. However, a Tg’ even higher than -36°C is desirable as it can shorten FD time, improve FD efficiency, and reduce costs, in addition to preserve the stability of drug products.

### Analytical methods

*Plaque assay (PA).* This potency assay was used to determine the infectious titer of a viral vaccine candidate. Summarily, Vero cell monolayers were infected with serially diluted samples. After a one-hour virus adsorption, culture media was added to the wells. After a four-day incubation at 34°C ± 1°C under 5% ± 2% CO_2_, the infected cells were fixed with methanol and immune-stained with an anti-viral monoclonal antibody conjugate (Envigo RMS Inc., IN. USA). Plaques were stained with a 3,3’,5,5’Tetramethylbenzidine (TMB)-blotting solution (Thermo Fisher, Canada). Each plaque was assumed to represent a single virus particle in the original sample, which was defined as one plaque forming unit (PFU). Plaques were visualized and quantified using a Viruscope™ (MicroVision Instruments; Evry, France) automated imaging system. The plaque count was used to determine the viral titer of the original sample in log_10_ PFU/mL. The internal control reference lots (Sanofi, ON. Canada) with known infectious titer ranging between 6.9 to 7.9 log_10_ PFU/mL was included in each plaque assay. Virus infectivity loss after the one-week accelerated stability study was calculated by subtracting titers after the one-week incubation from that of the original sample.

*Differential scanning calorimetry (DSC).* All Tg’ data were determined by using a power compensation DSC equipped with an Intracooler II (DSC8500; PerkinElmer LLC, Norwalk, CT, USA), as previously described [27]. An experimental coefficient of variation of 10% was estimated, leading to determination of Tg’ at ± 3ºC.

### Bayesian optimization algorithm

The ChemAssistant^®^ software (deepmatter^®^ Group Limited, Lyon, France) is the ML platform used in this study. It uses a BO algorithm to solve complex “black-box” optimization problems in as few experiments as possible. The BO approach uses two major design decisions.

The first one is a GP ML model. This type of model excels at estimating the uncertainty of its predictions *via* the variance of its function distribution [29]. More specifically, it models the data already acquired in previous steps of the optimization process and provides well-calibrated uncertainties for every prediction, which is crucial for the exploration strategy (choosing which areas to explore to improve model performance).

Firstly, a GP prior encodes assumptions that the uncertainty of function f at a given point x follows Gaussian probability distribution and represents an expectation over our belief about what the value of the function f is. An important property of a GP is that the marginal distribution of a finite number of variables (x) of a GP is a multivariate Gaussian distribution shown in Eq. 1:


$$f[x]\;\sim \;GP\left(\mu (x),\sigma^{2}(x)\right)$$ (1)

Where $\sigma^{2}(x)=K(x,x^{\prime })$, *µ* is the mean of the joint distribution and σ² or K(·, ·) represents the covariance matrix and is a kernel function that determines the covariance between any two function evaluation points. The covariance matrix is defined by the Matérn kernel covariance function using v = 5/2 yielding Eq. 2:


$$\begin{array}{c} K\left(x,x^{\prime }\right)=\left(1+\frac{\sqrt{5}\left|x-x^{\prime }\right|}{l}+\frac{{5\left(x-x^{\prime }\right)}^{2}}{{3l}^{2}}\right)exp\left(-\frac{\sqrt{5}\left|x-x^{\prime }\right|}{l}\right) \end{array}$$ (2)

where l is a positive constant that determines the characteristic length-scale of the process. The parameter v controls the level of smoothness of the function [30]. The distribution function at any new point

$x^{\prime }$ can be estimated given by the new mean *µ* and the uncertainty by the variance σ². Uncertainty is lower near observations and higher at a distance.

The second major design decision for BO is the choice of an acquisition function. The function determines the most interesting experiment(s) to perform in the next iteration, given the conditional distribution over the mean *µ*, and the variance σ². The ideal acquisition function should balance exploration against exploitation; that is, suggesting experiments that would improve the model accuracy versus experiments with high probability of finding an optimum based on the GP model’s optimal result predictions [31].

There exist different acquisition functions. However, the focus here will be on Lower Confidence Bound (LCB) defined by:


$$LCB\left(x^{\prime }\right)=\;\mu \left(x^{\prime }\right)-\kappa \cdot \sigma \left(x^{\prime }\right)$$ (3)

where κ 0 is an exploration-exploitation tradeoff parameter where lower κ is accompanied by BO favoring exploitation with function value near mean $\mu (x^{\prime })$ and higher κ favors exploration where uncertainty (or the posterior variance) is higher.

As such, the acquisition function suggests optimal settings for the next experiment. Over successive measurements and updates of the GP ML model, the GP surrogate model converges to the unknown objective function within the range of explored inputs, and the process can be continued until a satisfactory optimum is found.

### ML model generation and validation

**Fig 1:**
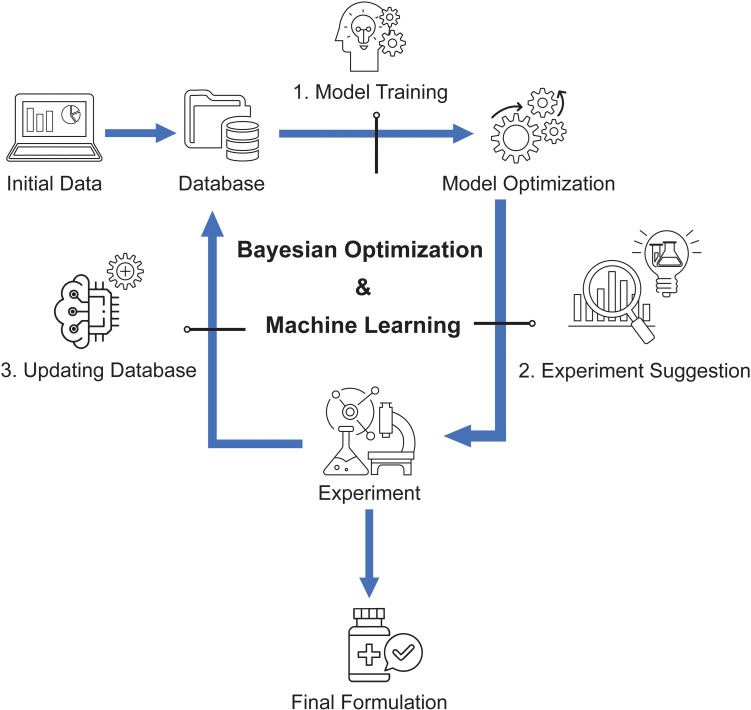
Schematic work-flow diagram of vaccine formulation development using machine learning-based tool.

The ChemAssistant^®^ platform generated a GP model, and then suggested new experiments using a balance between exploration (designed to improve the GP accuracy) and exploitation (designed to find solutions to the optimization problem) strategies. The results of the suggested experiments were integrated into the original dataset to refine the model and improve the quality of the suggestions. These steps of suggesting experiments using the Bayesian optimizer and updating the dataset were reiterated until the desired objectives were achieved. The assessments of variables’ permutation importance and Shapeley Additive exPlanations (SHAP) analysis helped to incrementally progress toward a final model with satisfactory predictive power, by removing non-influent model inputs. Fig 1 is a simplified workflow for illustrating ML application in formulation development. S1 File contains all terminology definitions.

In case study 1, the ML model was initially generated from historic data using one-week infectious titer loss at 37°C. To improve the model accuracy, additional data points supplemented from BO suggested additional experiments. Other empirically designed experiments were integrated for model optimization (S1 Table). Furthermore, feature selection was used to remove non-impactful variables. Additional reiterations and focused analysis were performed only on FOI to generate a model with satisfactory accuracy and cross-validation metrics. Extra trees regression model was used throughout the model optimization. The final model was validated using the holdout method by comparing prediction data against a test dataset comprising of experimental data never used in model generation (S2 and S3 Table). This is a validation step was performed in addition to the internal cross-validation examined by the software. Furthermore, prediction of the optimization zone for one FOI was plotted and visualized by using Python Matplotlib library (S4 Table).

In case study 2, the vaccine formulation optimization stage involved screening various excipients to maximize the glass transition temperature (Tg’). The nature and concentration of these excipients in the formulation affect the value of Tg’, which can be described by the Gordon-Taylor equation [32]. The ML model used in this study was developed using Tg’ data from 125 formulations, containing different excipients at various concentrations (S5 Table). To evaluate the selected model accuracy, a test set comprising 20 additional excipient combinations was employed.

## Results

### Model optimization virus A formulation case study 1

#### ML model generation and optimization.

For the case study 1, the ML approach consisted of 3 main stages: model training, model optimization, and model validation. Table 1 provides a comprehensive overview of key features utilized in the two first stages, presenting model training and optimization process in 5 steps.

**Table 1:** Data size, algorithm, and cross validation metrics in case study 1.

Step and objective	Data size	ML model type	Hyperparameters	Cross-validation metrics
Step 1: Model training from existing historical data to get the first-generation model	107	Extra trees	N_estimators = 30 Max_depth = 20	RMSE = 0.14 ± 0.02 R^2^ = 0.87
Step 2: Increase data set to improve predictions’ accuracy	128	Extra trees	N_estimators = 30 Max_depth = 20	RMSE = 0.15 ± 0.04 R^2^ = 0.9
Step 3: Feature reduction and introduction of new intrinsic variable	128	Extra trees	N_estimators = 30 Max_depth = 20	RMSE = 0.15 ± 0.02 R^2^ = 0.91
Step 4: Reiteration of suggested experiments to improve prediction accuracy	156	Extra trees	N_estimators = 30 Max_depth = 20	RMSE = 0.13 ± 0.02 R^2^ = 0.91
Step 5: Additional analysis to focus on a specific feature: rHSA	202	Extra trees	N_estimators = 30 Max_depth = 20	RMSE = 0.12 ± 0.01 R^2^ = 0.93

In step 1, a first set of 107 data points was used for the initial ML model training and included 19 features (excipients) from historical studies. The Extra trees algorithm was automatically selected with 30 trees and maximum tree depth of 20 by ChemAssistant® as the optimal ML model. The model cross-validation shows R^2^ = 0.87 and RMSE = 0.14 ± 0.02. This is a critical step of an appropriate prediction model training, as it demonstrates that the algorithm can successfully describe the dataset and can predict the desired output.

In step 2, 10 experiments suggested by the Bayesian optimizer using a balanced optimization strategy (mixing the exploration and exploitation strategies) were conducted and generated 21 data points. The total 21 new titer loss data points were combined with the original dataset, thus expanding the number of data points from 107 to 128. The model was re-trained, which resulted in an incremental improvement of R^2^ from 0.87 to 0.90 after step 1 was reiterated, indicating an improvement of the model’s predictions accuracy.

**Fig 2:**
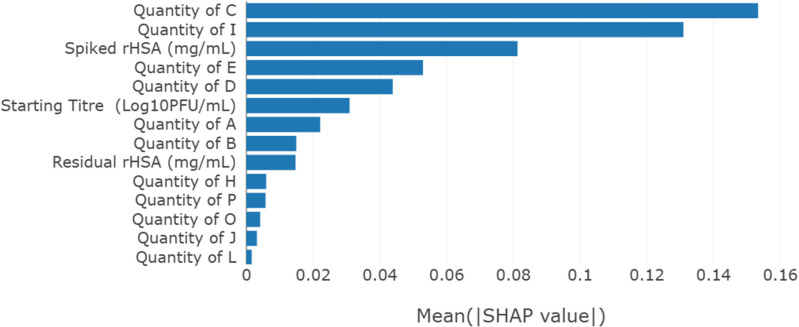
SHAP analysis used for feature selection in case study 1 step 3 analysis applying the selected Extra trees model for virus A titer loss after one-week at 37°C. SHAP values represent the contribution of each FOI to the model on its prediction. A SHAP value equal to zero means that that value of the input does not influence the predicted output relative to the average output value in the dataset.

In step 3, feature selection was undertaken by performing a SHAP analysis in the model generated in step 2. The features were ranked by SHAP values in a plot generated by ChemAssistant®, as shown in Fig 2. Out of 19 features (or excipients), 9 were impactful, and the model of the 9 FOI was generated, further improving R^2^ value from 0.90 to 0.91, and narrowing RMSE from 0.15 ± 0.04 to 0.15 ± 0.02. The increase of R^2^ value suggested that the 10 features removed from the model were indeed irrelevant attributes to the model quality.

In step 4, ChemAssistant^®^ suggested 5 experiments using a balanced exploration-exploitation strategy by BO algorithm and 10 additional data points were generated. The other 18 data points were generated by varying FOIs concentrations, in which spiked rHSA concentrations varied from 0 to 5.0 mg/mL and residual rHSA concentration was fixed at 1.2 mg/mL. Step 4 model showed an improvement in RMSE from 0.15 ± 0.02 to 0.13 ± 0.02.

Step 5 aimed to improve prediction accuracy for a specific FOI. The key feature was identified by Wrapper method with single-feature backward elimination. This process involved monitoring changes in model performance metrics from the model generated in step 4 as features were removed. The feature importance analysis is summarized in Table 2. rHSA was identified as one of the most impactful features and chosen as the FOI to challenge the model. This choice was made due to the projected non-linear response between titer losses and total rHSA concentrations, where residual and spiked rHSA are correlated features originating from different process steps. Residual rHSA is rHSA carried over from bioprocessing and spiked rHSA is the amount of rHSA addition as an excipient to a formulation. Together, they formed one feature as total rHSA. Given that residual rHSA also play a role in stabilization during the upstream and downstream bio-processing [33–35], Table 2 separates residual and spiked rHSA. The cross-validation metrics values for total, spiked, and residual rHSA demonstrate each feature’s influence on the prediction model. To further investigate the rHSA variable, 46 additional datapoints were generated from new experiments. The experiments used spiked rHSA concentration from 0 to 1.40 mg/mL and residual rHSA concentrations from 0.001 to 0.29 mg/mL. The rHSA concentrations for these new experiments were based on prior knowledge and available drug substance batches, using an empirical experimental design. The final model generated from 202 datapoints, showed an improved model performance with an increase of R^2^ value from 0.91 to 0.93 and a decrease of RMSE from 0.13 ± 0.02 to 0.12 ± 0.01.

**Table 2:** Analysis of feature importance by Wrapper method using single feature elimination.

Feature removed from full model	Data size	ML model type	Hyperparameters	Cross-validation metrics
Full Model	156	Extra trees	N_estimators = 30 Max_depth = 20	RMSE = 0.13 ± 0.02 R^2^ = 0.91
Excipient A	156	Extra trees	N_estimators = 30 Max_depth = 20	RMSE = 0.16 ± 0.03 R^2^ = 0.88
Excipient B	156	Extra trees	N_estimators = 30 Max_depth = 20	RMSE = 0.16 ± 0.04 R^2^ = 0.86
Excipient C	156	Extra trees	N_estimators = 30 Max_depth = 20	RMSE = 0.25 ± 0.07 R^2^ = 0.67
Excipient D	156	Extra trees	N_estimators = 30 Max_depth = 20	RMSE = 0.15 ± 0.03 R^2^ = 0.88
Excipient E	156	Extra trees	N_estimators = 30 Max_depth = 20	RMSE = 0.16 ± 0.04 R^2^ = 0.86
Excipient I	156	Extra trees	N_estimators = 30 Max_depth = 20	RMSE = 0.19 ± 0.03 R^2^ = 0.81
Starting Titer	156	Extra trees	N_estimators = 30 Max_depth = 20	RMSE = 0.18 ± 0.07 R^2^ = 0.81
Spiked rHSA + Residual rHSA (Total rHSA)	156	Extra trees	N_estimators = 30 Max_depth = 20	RMSE = 0.24 ± 0.08 R^2^ = 0.68
Spiked rHSA	156	Extra trees	N_estimators = 30 Max_depth = 20	RMSE = 0.17 ± 0.02 R^2^ = 0.85
Residual rHSA	156	Extra trees	N_estimators = 30 Max_depth = 20	RMSE = 0.2 ± 0.04 R^2^ = 0.79

#### Model inspection and functionality check.

**Fig 3:**
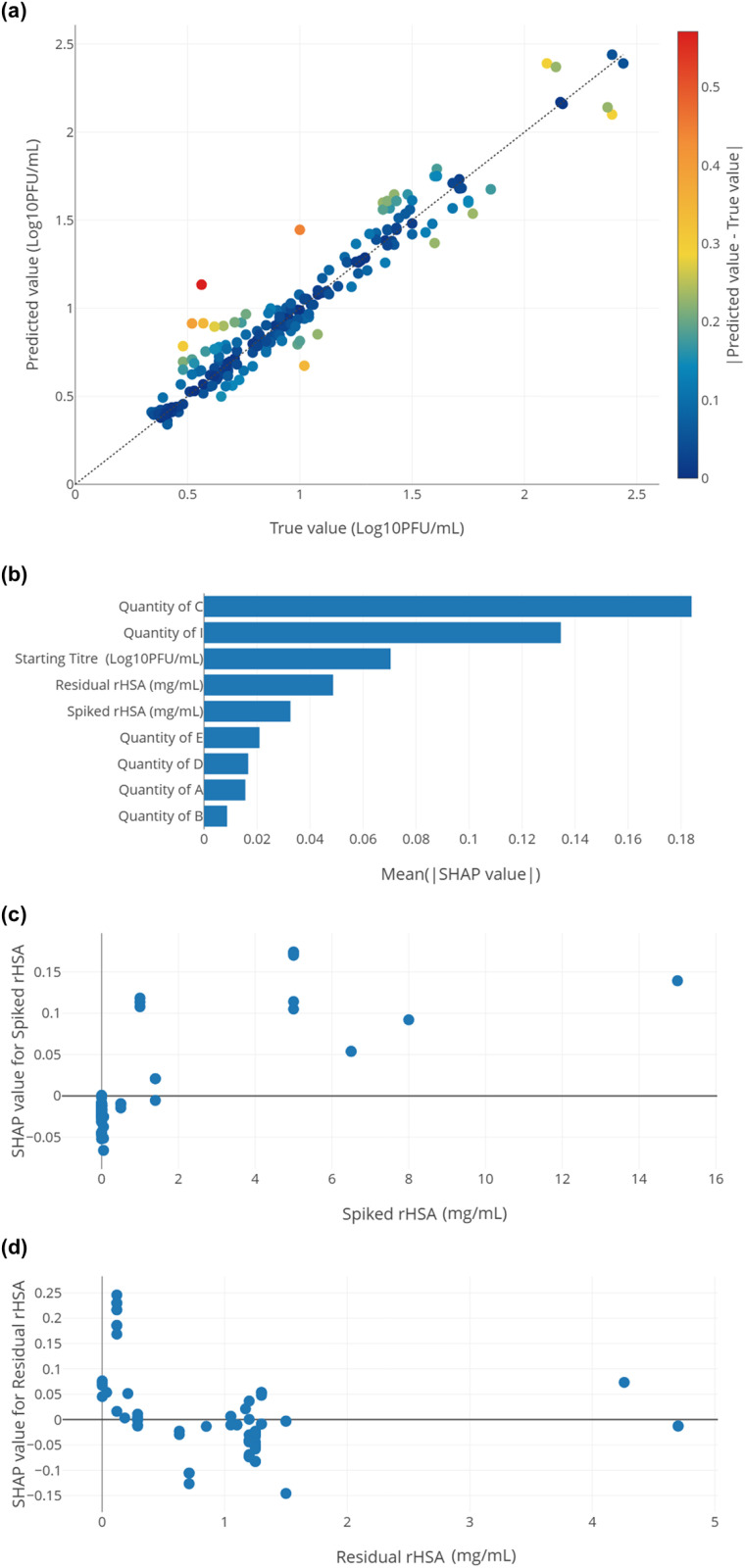
Details on excipient of interest in case study 1 step 5 analysis applying the selected Extra trees model for virus A titer loss following a one-week incubation at 37°C. **(a)** Prediction error plot of titer losses. R^2^ = 0.93. Each experimental data point is colored based on the distance between predicted and experimental data; blue and red represent small and large differences, respectively. **(b)**: Global SHAP analysis of all features summarizing the influence of each FOI on predicted SHAP mean value. **(c)**: SHAP analysis of spiked rHSA. **(d)**: SHAP analysis of residual rHSA. A positive SHAP value means that the input increases the predicted output relative to the average output value in the dataset (i.e., higher predicted titer loss predicted by the model), and vice-versa for a negative SHAP value.

Fig 3 shows the model inspection, indicating which model inputs were impactful, and how these model inputs influenced the predicted output. Fig 3(a) shows that the model’s error is homogeneously distributed. Fig 3(b) shows that the most influent variables in the titer loss model are the Quantity of C and Quantity of I, followed by the Starting titer, residual rHSA, and spiked rHSA. Other variables have a weaker (but non-null) influence on the model. Fig 3(c) shows that an increased amount of spiked rHSA is correlated with a higher titer loss predicted by the model. The correlation is non-linear and a spiked rHSA concentration higher than 2 mg/mL does not further increase the predicted titer loss in the model. Fig 3(d) shows that a residual rHSA concentration lower than 0.2 mg/mL is correlated with a higher titer loss.

#### ML model validation.

To evaluate the prediction model, we compared the experimentally determined one-week titer losses at 37°C with model predicted values using the holdout method (see Table 3). The model was tested by using a test set, consisting of 22 new experimental data points that were never used in any steps of model development. These 22 datapoints in the test set represented formulations with varying quantities of the 9 influential features identified in step 3.

**Table 3:** Model validation (case 1).

	Model used	Data Included in model generation	Model Prediction Accuracy
**Test Set**	Step 3	No	50% (11/22)
	Step 4	No	63.6% (14/22)
	Step 5	No	86.4% (19/22)

Assessing the performance of step 3 and 4 models, the accuracy of titer loss predictions was 50% (11 correct predictions out of 22 datapoints) and 63.6% (14 correct predictions out of 22 datapoints), respectively. In contrast, predictions using same test dataset yielded 86.3% correct predictions (19 correct predictions out of 22) with step 5 model.

### Model predicting Tg’ of a complex virus B formulation in case study 2

**Fig 4:**
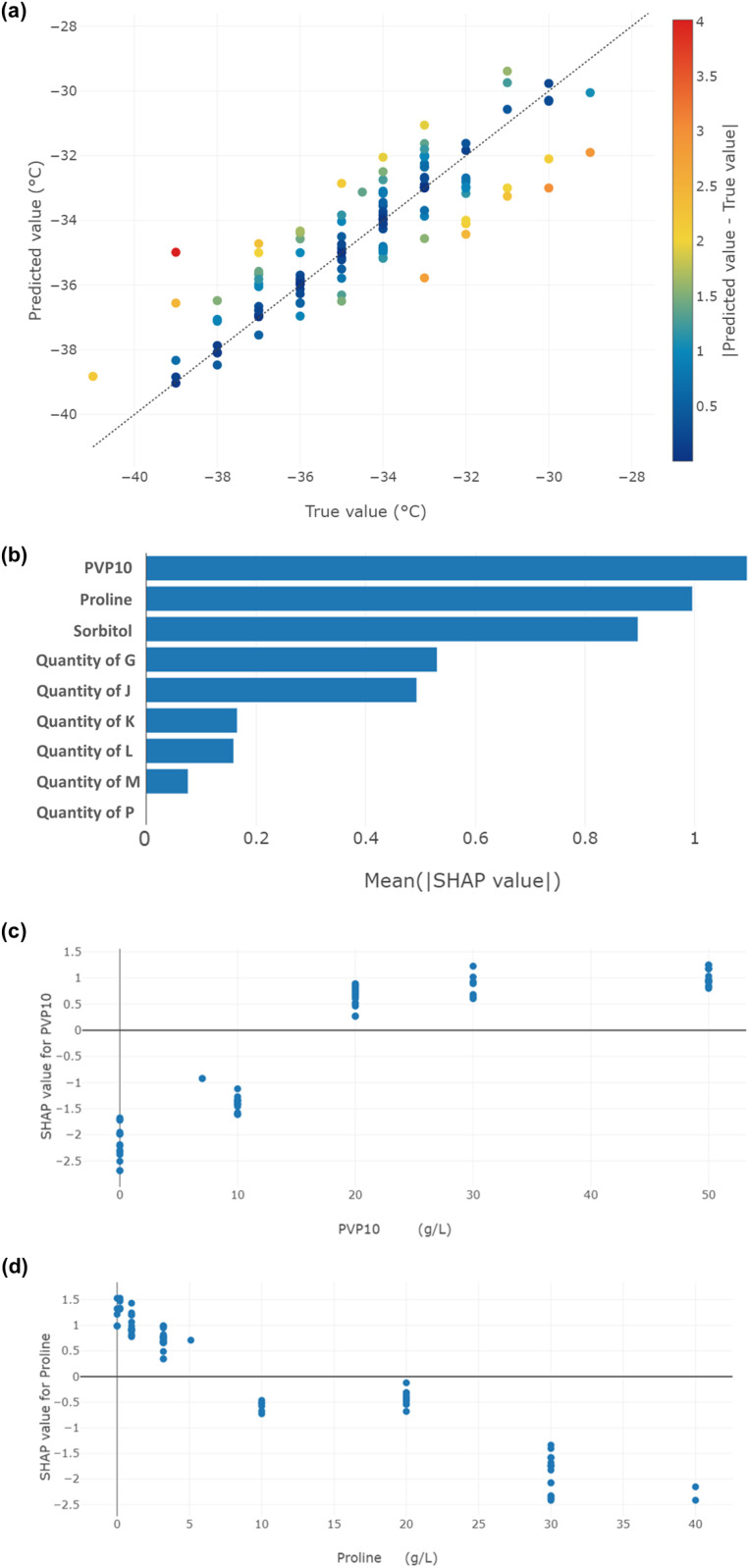
Details on excipient of interest analysis in case study 2 applying the selected XGBoost model for Tg’ predictions. **(a)** Prediction error plot. **(b)** Global SHAP analysis of all features **(c)** SHAP analysis of PVP10. **(d)** SHAP analysis of proline.

During the development of virus B vaccine formulation for a Phase I clinical trial, a broad set of Tg’ data points were generated. A total of 125 Tg’ values obtained from formulations based on nine selected excipients, including a buffer, two amino acids, a polysaccharide, a polyol, and a polymer, to build the model. The best selected model for this case was an XGBoost model, which demonstrated good cross-validation metrics: RMSE = 1.17 ± 0.17, R² = 0.75. In Fig 4(a), the prediction error plot shows no abnormal distribution or outliers in this model. The global SHAP plot (Fig 4(b)) highlighted significant impact of PVP, proline and sorbitol. PVP is known to increase the Tg’ (-26°C) [36]. Its positive impact was confirmed by the selected model, as shown by its SHAP diagram (Fig 4(c)). Conversely, proline and sorbitol were expected to have a negative impact on Tg’ [37]. The selected model and SHAP diagrams (Fig 4(d) for proline) also confirmed this negative impact.

After confirming the reliability of the model, 20 additional data points, not used to build/train the model and covering the Tg’ range from -37°C to -31°C, were selected to test the model. All Tg’ obtained from the model were accurate, with the average and maximum error of 0.33°C and 1.88°C, respectively. This was in good agreement with Tg’ determination by DSC.

**Fig 5:**
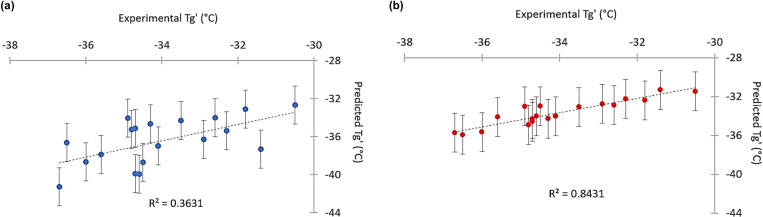
Predicted Tg’ errors for 20 additional formulations of case study 2 using linear (a) and XGBoost (b) models. (a) Experimental vs predicted Tg’ for Linear (a) panel in blue and XGBoost models (b) panel in red. Dotted lines to guide eye.

For comparison, a linear model was tested. Although this simpler model exhibited acceptable statistical metrics (RMSE = 1.31, R² = 0.69), the predicted Tg’ values were predominantly underestimated, with errors reaching up to 6°C (Table 4, Fig 5).

**Table 4:** Experimental Tg’ of 20 additional formulations compared with predicted Tg’ using XGBoost and linear models.

		Linear Model			XGBoost Model	
Formulation	Experimental Tg’ (°C)	Predicted Tg’ (°C)	Predicted error/delta (°C)	Predicted Tg’ (°C)		Predicted error/delta (°C)
#1	−36.0	−38.7	−2.7	−35.7		0.3
#2	−34.1	−37.0	−2.9	−34.0		0.1
#3	−36.7	−41.3	−4.6	−35.7		1.0
#4	−34.6	−40.0	−5.4	−34.0		0.6
#5	−34.5	−38.7	−4.2	−33.0		1.5
#6	−32.9	−36.3	−3.4	−32.8		0.1
#7	−34.8	−35.3	−0.5	−34.9		−0.1
#8	−34.3	−34.7	−0.4	−34.3		0.0
#9	−31.4	−37.3	−5.9	−31.3		0.1
#10	−30.5	−32.7	−2.2	−31.5		−1.0
#11	−36.5	−36.7	−0.2	−35.9		0.6
#12	−34.1	−37.0	−2.9	−34.0		0.1
#13	−34.9	−34.1	0.8	−33.0		1.9
#14	−32.3	−35.4	−3.1	−32.3		0.0
#15	−34.7	−39.9	−5.2	−34.6		0.1
#16	−35.6	−37.9	−2.3	−34.1		1.5
#17	−31.8	−33.1	−1.3	−32.4		−0.6
#18	−32.6	−34.0	−1.4	−32.9		−0.3
#19	−34.7	−35.1	−0.4	−34.3		0.4
#20	−33.5	−34.3	−0.8	−33.1		0.4

## Discussion

### Model generation and optimization for case 1

**Fig 6:**
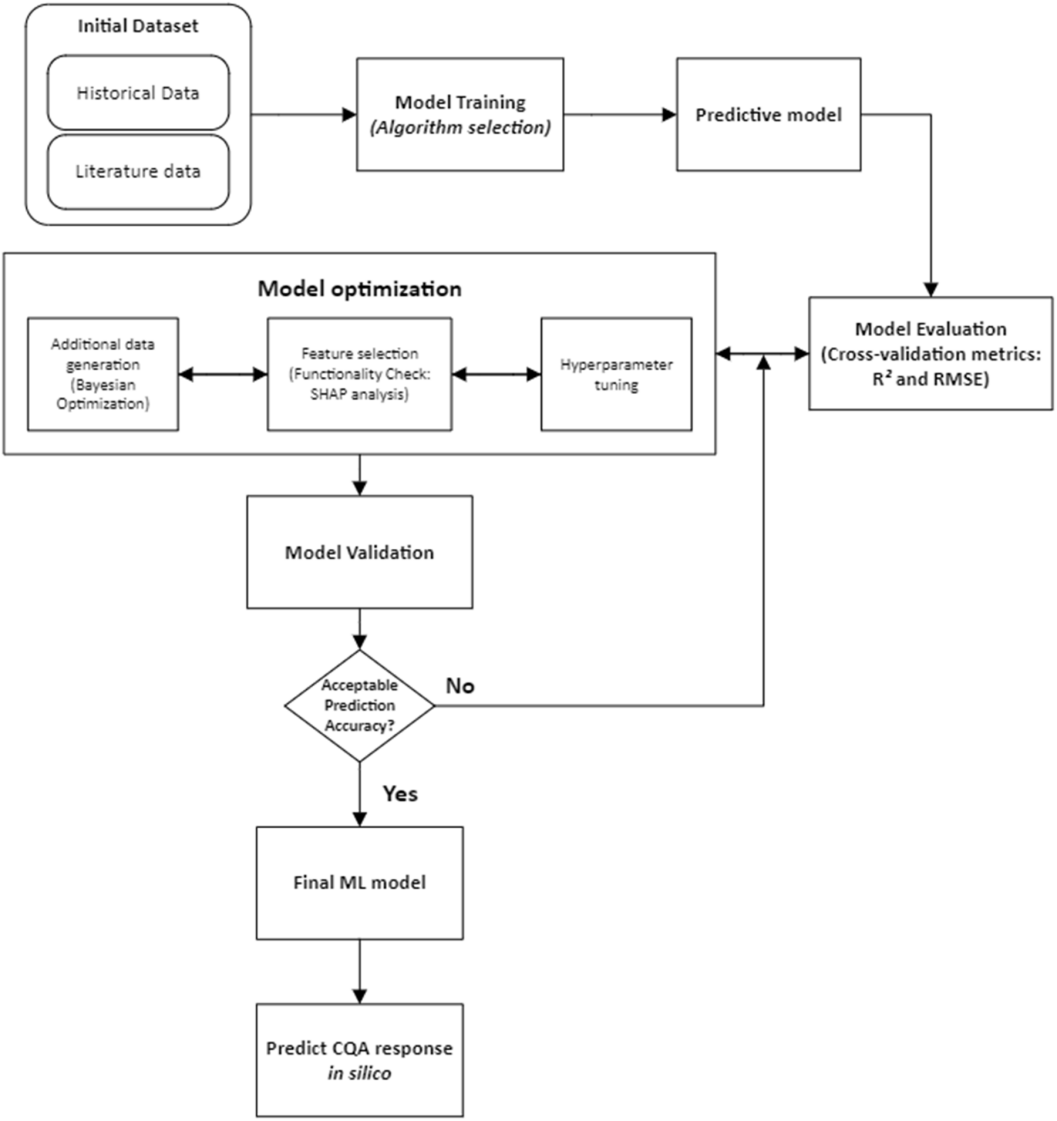
Model optimization strategy and decision tree.

As illustrated in Fig 6, the general strategy to obtain a high accuracy model uses BO to iteratively suggest new experiments in the areas of higher uncertainty. The results of these new experiment are then integrated into the dataset, progressively improving the model accuracy.

Table 1 presents the details of the ML prediction models that have been generated and optimized. These models achieved R^2^ values ranging from 0.87 to 0.93, and RMSE values from 0.14 ± 0.02 to 0.12 ± 0.01. All models used fixed hyperparameters: 30 decision trees and 20 maximum depths of the trees. Hyperparameter tuning was not performed in our case 1 to isolate the effect of model optimization. It also demonstrated that ML can generate prediction models for a specific output, in this case, is the titer loss after one-week incubation at 37°C. The model’s R^2^ value provides insight into the quality of the prediction model, it representing the “goodness-of-fit” between the predicted values and the true experimental values. The prediction error plot, Fig 3(a), offers a visual estimation of this fit and the correlation between predicted and true values. In combination with the global SHAP analysis in Fig 3(b), these tools enable scientists to estimate which excipient has greater impact on the vaccine formulation stability. The wrapper method analysis in Table 2 suggests that total rHSA has a major influence on model accuracy, with residual rHSA having a greater impact than spiked rHSA. This is aligned with the individual SHAP analysis in Fig 3(c) and 3(d). Fig 3(c) shows that spiked rHSA concentrations from 1.0 mg/mL up to 15.0 mg/mL have a more substantial impact, leading to higher titer loss. In contrast, Fig 3(d) projects that the concentrations of residual rHSA from 0.2 to 1.5 mg/mL have a greater impact in minimizing titer loss, corresponding to improved formulation stability. This insight guided us to maintain minimum concentration of residual rHSA at 0.2 mg/mL. These analyses highlight the non-linear relationship between rHSA concentration and titer loss.

To further understand each excipient’s impact on model accuracy, a feature importance analysis compared to full features using a single feature elimination Wrapper method was performed. The results are summarized in Table 2. This analysis involved removing one feature at a time and then assessing the model performance through its R^2^ value. The greater change in R^2^ value represents a stronger influence of the removed feature on model accuracy. From Step 4, it was found residual rHSA had more influence on formulation stability than spiked rHSA, as evidence by larger decrease in R^2^. Given that the roles of residual and spiked rHSA were not well understood and characterized in early development, a new model was optimized (step 5 in Table 1). This model incorporated additional data from various rHSA concentration studies while keeping other excipients and their concentrations constant. This optimization further advanced the model accuracy, increasing R^2^ to 0.93. This focused analysis solidified our understanding of rHSA’s role as a stabilizer and revealed the criticality of residual rHSA carried over from the drug substance bioprocess. The functions of rHSA in the bioprocess have been discussed in literatures [33–35], highlighting its multi-functional roles. These include working as a physical shear protectant, and antioxidant, all of which contribute to process and product consistency. For context, typical rHSA amounts in licensed live attenuated virus vaccines are 0.3 mg per dose (0.5 mL) in M-M-R II [38], 2% in ACAM2000 [39].

**Fig 7:**
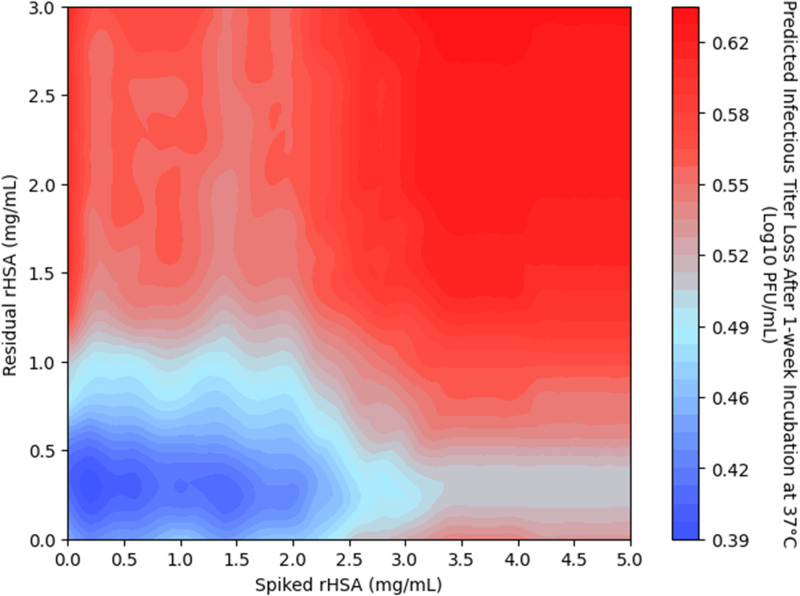
Correlation heatmap showing non-linear correlation of residual rHSA and spiked rHSA concentration to the predicted titer loss at one-week 37°C.

The heatmap in Fig 7 was generated using the prediction model to visualize the non-linear correlation between residual rHSA and spiked rHSA concentration and the titer loss. An optimal zone with lower titer losses can be identified to serve as guidance for process and product quality control. For case 1, the zone has the boundary of residual rHSA concentrations from 0.05 to 0.6 mg/mL and spiked rHSA concentrations from 0 to 1.5 mg/mL. Based on this model, the preferred area would be 0.2–0.4 mg/mL for residual rHSA and 0–0.6 mg/mL for spiked rHSA, respectively. Within this zone, and with fixed concentrations of other excipients, the vaccine formulation would experience the lowest titer loss and the best thermal stability.

Validation of the optimized models from step 3–5 was done by comparing previously unused experimental data against the model prediction data (Table 3). At each step of model optimization, there was a progressive improvement in prediction accuracy, increasing from 50% to 86%. This improvement demonstrates the significancy of feature importance analysis and the value of adding data in areas of high uncertainty to enhance model performance. The acceptable accuracy of the final predictive model warranted no further optimization.

### Model generation and comparison for case 2

Case study 2 focused on predicting the Tg’ values of vaccine formulations. Traditionally, empirical equations, namely the Gordon-Taylor and Fox equations, have been used to predict glass transition temperatures considering Tg’ and the respective proportions of each excipient in a formulation of interest [37]. However, these equations were primarily tested on simple binary amorphous systems and often showed deviations between predicted and experimental glass transition temperatures. This is due to the interactions in between the formulation components [37]. In contrast, ML models such as XGBoost selected for case study 2 can model non-linear responses surfaces, leading to more accurate prediction of Tg’.

In summary, the high accuracy rates achieved in our studies underscored the strong capabilities of BO in modeling and predicting the performance of complex vaccine formulation. The approach successfully leverages data generated throughout all cycles of vaccine development, including historical data from early phase development, intermediate development data from bioprocess modifications and formulation screening, and data from late phase development.

### Considerations of Bayesian optimization and ML models

The conventional DoE uses controlled design with purposely selected factors and levels to statistically optimize experiments. The objective is to construct simple models identifying relationships between variables and responses, often tailored for linear responses as well as dedicated unit operations for custom design. In contrast, ML, bolstered by rapid growth in data science, is better suited to capture complex processes and non-linear relationships due to its learning ability. ML can utilize all available data throughout development, regardless of when and how it was generated, focusing on building predictive models. From the two cases presented in this paper, BO can be a complementary tool to conventional DoE and empirical “trial-and-error” approaches. BO employs an active learning strategy to suggest new exploratory experiments, select features, cross-validate, and optimize models [12]. The BO algorithm can effectively converge to global optimization objectives, particularly beneficial when available data and experimental budget are limited [40,41], or reveal the non-linear relationships unforeseen in the empirical and DoE experiments.

Bayesian optimization exploitation strategy proposes new formulations predicted by the GP, trained on existing data, to give optimal results. Conversely, the exploration strategy suggests formulations distinct from the known data, aiming to bridge knowledge gaps and increase the GP accuracy. To achieve a good optimization performance, it is necessary to balance exploitation and exploration. The weight of these strategies in any project depends on the global objectives, prior product knowledge, and available resources to execute given suggestions from the Bayesian optimizer. However, variable constraints should be carefully determined during modeling to avoid nonsensical experiments proposed by the Bayesian optimizer (e.g., concentration of an excipient that exceeds its solubility limits, or an extreme pH value incompatible with live viruses). To improve the model’s performance, dataset curation and feature selection may be necessary to focus on the most influential variable(s).

Overall, BO modeling is a powerful tool that can provide qualitative and quantitative inputs and insights for complex vaccine formulation development. It helps scientists optimize vaccine formulations by maximizing the value of existing data and minimizing the development costs through predictive modeling. With iterative experiments and model refinement, it becomes possible to predict responses accurately and narrow down investigations to a few optimized formulations, regardless of the formulation matrix complexity, the multiple degradation pathways, the raw material sources, and the batch or assay variations. SHAP analysis, permutation importance analysis, and model evaluation enable scientists to analyze the impacts of excipients on stability in complex formulations.

Recently, more biopharmaceutical companies have committed strategically to digital AI/ML transformation. Sanofi announced an “all in” approach to artificial intelligence and data science to accelerate breakthroughs for patients [42]. Merck invested in an “end-to-end” AI platform to “fast-track the development of new and truly innovative candidates [43], and many more examples exist. The FDA has also published an open discussion paper [44] addressing various aspects and considerations of using AI or ML in drug and biological product development. ML represents a new frontier for vaccine formulation development, requiring alignment with global regulatory bodies’ expectations [42–45]. Building a healthy and responsible ecosystem for ML and data science integration within the pharmaceutical industry is crucial.

## Conclusion

AI and ML are shifting the paradigm of biological development by removing bottleneck in vaccine research and development [46–48]. The rapid growth of data science and advanced computational technologies offers tremendous potential to accelerate development, enhance effectiveness, and improve the safety of vaccine products. In case study 1, Bayesian optimization demonstrated how to maximize the value of experimental data using ML models. Functionality check such as prediction error plot, SHAP analysis, permutation importance, and comparisons between algorithm-predicted values and actual experimental results were employed. Stepwise analysis and model optimization showed progressive improvements in model outcomes and prediction accuracy. Validation against a test dataset proved the model’s reliability. Furthermore, this analysis revealed the effects and optimal concentration range of rHSA. Using heatmaps, the predicted optimal zones for rHSA concentration were recommended. Such ML prediction outcomes, visualized through heatmaps or other methods, are useful for probing non-linear responses. Case study 2 showcased an ML model that accurately predicted glass transition temperatures of virus B vaccine formulations, incorporating seven excipients in various concentration ranges.

Together, these case studies demonstrate that an approach based on BO and ML modeling can be applicable to various types of CQAs in vaccine formulation development. This approach aligns well with the quality by design (QbD) principles [49,50] promoted by international guidelines throughout Chemistry, Manufacturing and Control (CMC) readiness for clinical phases [51]. It provides scientists with complementary methods to extract additional information from available experimental data, thereby enriching product knowledge.

Looking ahead, it would be valuable if future ML tools could offer additional model explanations, further opening the “black/grey box” to visualize relationships and interactions between features/responses and formulation/process parameters. This, in turn, would help scientists propose mechanistic descriptions of the underlying physical, chemical, or biological phenomena, serving process and product development with better data-driven decisions [52].

## Supporting information

S1 File Terminology Definitions. (DOCX)

S1 Table Data used for case 1 model generation. (PDF)

S2 Table Residual rHSA and spiked rHSA values for the test dataset in case 1. True experimental value of infectious titer loss for each study were compared against ML predicted values for infectious titer loss (log_10_ PFU/mL) using model generated in step 3–5. The test dataset is comprised of 10% data points used in the generation of the model.
(PDF)

S3 Table Residual rHSA and spiked rHSA values for the holdout dataset in case 1. True experimental value of infectious titer loss for each study were compared against ML predicted values for infectious titer loss (log_10_ PFU/mL) using model generated in step 3–5. The holdout dataset is comprised of 22 data points not used in the generation of the model (i.e., unforeseen data).
(PDF)

S4 Table Data used for generating Fig 7 heatmap prediction. (PDF)

S5 Table Data used for case 2 model generation. (PDF)
