# Supplementary material for: Bayesian optimization and machine learning for vaccine formulation development
Source: PLoS One. 2025 Jun 11;20(6):e0324205. doi: 10.1371/journal.pone.0324205 (PMC12157168; doi:10.1371/journal.pone.0324205)
Supplement: S1 File — (DOCX) [file pone.0324205.s001.docx]

**Terminology Definitions**

**Constraint:** The boundaries of the design space, defined based on prior knowledge. Constraints are used to restrict the parameter values of the experiments that are suggested by the optimization algorithm: the algorithm will only suggest experiments that verify the constraints equations.

**Bayesian Optimization**: Bayesian optimization is a machine learning-based optimization method. It is especially suitable for the optimization of black-box functions, of which the evaluation is expensive. Its first step is to build a Gaussian Process model from the available data. This Gaussian Process is based on a prior probability distribution, which is combined with the data (used to obtain the covariance matrix with the help of a kernel, chosen to be the Matérn kernel, in our case) to build the posterior distribution. This posterior distribution can be sampled at any point to obtain a predicted value (the mean of the Gaussian Process distribution) and a confidence interval (based on the standard deviation of the Gaussian Process distribution). The next step of the Bayesian Optimization algorithm is to apply an acquisition function to the Gaussian Process model. In our case, we used the Lower Confidence Bound acquisition, with an alpha coefficient equal to 5. Then, the optimum of the acquisition function is searched using a multi-started gradient descent. That optimum corresponds to the next experiment to be performed. That experiment is performed, its result is added to the dataset, the Gaussian Process can be updated, and the Bayesian Optimization algorithm can continue. Typically, the Bayesian Optimization algorithm continues until a satisfactory optimum is found or the maximum number of experiments defined by the user is reached.

**Permutation Importance:** A technique to estimate the importance of each factor of a ML model. It works by measuring the difference between the predictive power (measured by the R^2^) of a baseline model (trained on the original dataset) and a model trained on a dataset inside which the values of one of the factors have been randomly permuted. The higher that difference, the higher the permutation importance, meaning that the factor is of high importance in the model.

**SHAP (Shapeley Additive exPlanations):** SHAP analysis is a mathematical method that can be used to assess the contribution of variables on the output of the model [30]. SHAP values represent the contribution of each factor of the model on its prediction, assuming that the contributions of all factors are additive. In ChemAssistant^®^, SHAP values are calculated for a random sample of 100 data points of the dataset (or all data points, if there are fewer than 100 data points in the dataset). For each model input, the SHAP value of each of the 100 data points is plotted against the value of the given model input. A SHAP value equal to zero means that that value of the input does not influence the predicted output relative to the average output value in the dataset. A positive SHAP value means that the input increases the predicted output relative to the average output value in the dataset (i.e. higher predicted titer loss predicted by the model), and vice-versa for a negative SHAP value.

**Cross-validation Metrics:** A way of measuring the quality of predictions made by a ML model on points that have not been used for model training. ChemAssistant^®^ uses 5-fold cross-validation: the dataset is randomly split in 5 portions of 20%, then for each portion, a ML model is trained on the 80% remaining data, then tested on the 20% portion. The operation is repeated 5 times (once for each 20% portion) and the metrics are aggregated over the 5 folds.

**Root Mean Square Error (RMSE):** Represents the standard deviation (SD) of the residuals (prediction errors) and is used to measure the extent to which model predictions deviate from the data points. In other words, RMSE is a measure of the average distance between the predicted value of a model and the actual experimental value. This metric is commonly used to evaluate model prediction accuracy. Larger RMSE values indicate a poor model quality while an RMSE equal to zero implies a perfect fit between the model predictions and the actual dataset.

**Exploitation**: An optimization strategy that trusts the predictions of the model that is built from the data and goes where good results (according to the objectives) are predicted.

**Exploration:** An optimization strategy in the zones of experimental space where the model uncertainty is high. It improves the Gaussian Process model by reducing its uncertainty in these zones. It also prevents the optimization algorithm from getting stuck on local optima.

**Prediction:** to test your hypotheses or understand your data (i.e. results of previous experiments) by building and using/inspecting a predictive model. ChemAssistant™ includes a range of classification and regression models. Classification models can predict categorical variables, while regression models can predict numerical variables.

**Extra Tree Model:** An ensemble model, based on decision trees, in which the features and splitting thresholds are randomized for each tree.

**Random Forests Model:** A regression or classification machine learning (ML) model composed of an ensemble of decision trees. Each tree of the forest is built from a data sample drawn randomly with replacement from the training dataset. The random sampling makes each tree slightly different from the other trees, improving the predictive ability of the model. While building the trees, each node is split in order to minimize the Mean Square Error of the tree. The process stops when the tree has reached a predefined maximum depth or that that no leave can be further split. Predictions from the Random Forest are obtained by taking the mean of the predictions of all trees (respectively the mode of the predictions, for a classification model).

**XGBoost Model:** eXtreme Gradient Boosting, an optimized distributed gradient boosting library designed for efficient and scalable training of machine learning models.

**Hyperparameters:** a hyperparameter is a parameter that influences the structure of the ML model or the way the ML model learns. Hyperparameters are chosen before training the ML model. For example, in a Random Forest model, the number of trees or the maximum depth of the trees are two important hyperparameters. When training a model from a dataset, it is a common practice to try several hyperparameter values in order to maximize the model performance.

**Wrapper Method:** A feature selection method used to evaluate the impact or significance of a feature on a model based on model performance metrics. In backward elimination, a feature is removed from the full model and the change in model cross-validation metrics is evaluated to determine the “usefulness” of the feature. Large decrease in model performance metrics indicates that the feature is significant in predictive accuracy of the model.
