# Supplementary material for: Bayesian optimization and machine learning for vaccine formulation development
Source: PLoS One. 2025 Jun 11;20(6):e0324205. doi: 10.1371/journal.pone.0324205 (PMC12157168; doi:10.1371/journal.pone.0324205)
Supplement: S1 Table — (PDF) [file pone.0324205.s002.pdf]

S1 Table: Data used for case 1 model generation.

Experiment id	Dataset used in model generation - LL	Titer loss at 1 week 37C (Log10PFU/mL)	Residual rHSA (mg/mL)	Spiked rHSA (mg/mL)	Quantity of A	Quantity of B	Quantity of C	Quantity of D	Quantity of E	Quantity of F	Quantity of G	Quantity of H	Quantity of I	Quantity of J	Quantity of K	Quantity of L	Quantity of M	Quantity of O	Quantity of P	Quantity of Q	Quantity of R	Starting Titre (Log10PFU/mL)
Study 1-1	Step 1 model generation	0.79	5.41	0	40	10	160	0	0	0	0	0	100	0	0	0	0	0	0	0	0	7.12
Study 1-2		0.67	5.41	0	40	10	160	0	0	0	0	0	100	0	0	0	0	0	0	0	0	7.12
Study 1-3		0.48	0.71	0	40	10	160	0	0	0	0	0	100	0	0	0	0	0	0	0	0	6.23
Study 1-4		0.57	0.71	0	40	10	160	0	0	0	0	0	100	0	0	0	0	0	0	0	0	6.29
Study 1-5		1	4.26	0	40	10	160	0	0	0	0	0	100	0	0	0	0	0	0	0	0	6.77
Study 1-6		1	4.26	0	40	10	160	0	0	0	0	0	100	0	0	0	0	0	0	0	0	6.73
Study 1-7		0.91	1.32	0	40	10	160	0	0	0	0	0	100	0	0	0	0	0	0	0	0	6.21
Study 1-8		0.96	1.32	0	40	10	160	0	0	0	0	0	100	0	0	0	0	0	0	0	0	6.26
Stduy 2-1		1.43	1.25	0	40	10	0	0	0	0	0	0	0	0	0	0	0	0	0	0	0	6.98
Stduy 2-2		1.42	1.25	0	40	10	0	0	0	0	0	0	0	0	0	0	0	0	0	0	0	6.88
Stduy 2-3		1.38	1.25	0	40	30	0	0	0	0	0	0	0	0	0	0	0	0	0	0	0	6.96
Stduy 2-4		1.41	1.25	0	40	30	0	0	0	0	0	0	0	0	0	0	0	0	0	0	0	6.92
Stduy 2-5		0.93	1.25	0	40	10	80	0	0	0	0	0	0	0	0	0	0	0	0	0	0	7.13
Stduy 2-6		0.96	1.25	0	40	10	80	0	0	0	0	0	0	0	0	0	0	0	0	0	0	7.13
Stduy 2-7		0.68	1.25	0	40	10	160	0	0	0	0	0	0	0	0	0	0	0	0	0	0	7.11
Stduy 2-8		0.64	1.25	0	40	10	160	0	0	0	0	0	0	0	0	0	0	0	0	0	0	7.09
Stduy 2-9		0.87	1.25	0	40	10	0	0	0	0	0	0	50	0	0	0	0	0	0	0	0	7.06
Stduy 2-10		0.88	1.25	0	40	10	0	0	0	0	0	0	50	0	0	0	0	0	0	0	0	7.08
Stduy 2-11		0.68	1.25	0	40	10	0	0	0	0	0	0	100	0	0	0	0	0	0	0	0	7.06
Stduy 2-12		0.64	1.25	0	40	10	0	0	0	0	0	0	100	0	0	0	0	0	0	0	0	7.08
Stduy 2-13		0.66	1.25	0	40	10	80	0	0	0	0	0	50	0	0	0	0	0	0	0	0	7.09
Stduy 2-14		0.62	1.25	0	40	10	80	0	0	0	0	0	50	0	0	0	0	0	0	0	0	7.11
Stduy 2-15		0.62	1.25	0	40	10	160	0	0	0	0	0	100	0	0	0	0	0	0	0	0	7.08
Stduy 2-16		0.63	1.25	0	40	10	160	0	0	0	0	0	100	0	0	0	0	0	0	0	0	7.11
Stduy 2-17		1.34	1.25	0	40	10	0	5	0	0	0	0	0	0	0	0	0	0	0	0	0	7.07
Stduy 2-18		1.34	1.25	0	40	10	0	5	0	0	0	0	0	0	0	0	0	0	0	0	0	7.06
Stduy 2-19		1.44	1.25	0	40	10	0	10	0	0	0	0	0	0	0	0	0	0	0	0	0	7.04
Stduy 2-20		1.5	1.25	0	40	10	0	10	0	0	0	0	0	0	0	0	0	0	0	0	0	7.05
Stduy 2-21		1.02	1.25	0	40	10	0	0	5	0	0	0	0	0	0	0	0	0	0	0	0	7.06
Stduy 2-22		1.05	1.25	0	40	10	0	0	5	0	0	0	0	0	0	0	0	0	0	0	0	7.06
Stduy 2-23		1	1.25	0	40	10	0	0	0	0	0	10	0	0	0	0	0	0	0	0	0	6.98
Stduy 2-24		1.37	1.25	0	40	10	0	0	0	0	0	10	0	0	0	0	0	0	0	0	0	7.02
Stduy 2-25		1.39	1.25	0	40	10	0	0	0	0	10	0	0	0	0	0	0	0	0	0	0	6.95
Stduy 2-26		1.49	1.25	0	40	10	0	0	0	0	10	0	0	0	0	0	0	0	0	0	0	7
Stduy 2-27		1.4	1.25	0	40	10	0	0	0	10	0	0	0	0	0	0	0	0	0	0	0	7.03
Stduy 2-28		1.37	1.25	0	40	10	0	0	0	10	0	0	0	0	0	0	0	0	0	0	0	7
Study 3-1		1.71	1.25	0	40	10	0	0	0	0	0	0	0	0	0	0	0	0	0	0	0	6.87
Study 3-2		1.59	1.25	0	40	10	0	0	0	0	0	0	0	0	0	0	0	0	0	0	0	6.82
Study 3-3		0.89	1.25	0	40	10	250	0	0	0	0	0	0	0	0	0	0	0	0	0	0	7.14
Study 3-4		0.9	1.25	0	40	10	250	0	0	0	0	0	0	0	0	0	0	0	0	0	0	7.14
Study 3-5		1.39	1.25	0	45	10	0	0	0	0	0	0	0	0	0	0	0	0	0	0	0	7.04
Study 3-6		1.31	1.25	0	45	10	0	0	0	0	0	0	0	0	0	0	0	0	0	0	0	6.88
Study 3-7		0.66	1.25	0	45	10	160	0	0	0	0	0	0	0	0	0	0	0	0	0	0	7.09
Study 3-8		0.67	1.25	0	45	10	160	0	0	0	0	0	0	0	0	0	0	0	0	0	0	7.12
Study 3-9		1.42	1.25	0	40	10	0	0	0	0	0	0	0	0	0	0	1	0	0	0	0	6.96
Study 3-10		1.39	1.25	0	40	10	0	0	0	0	0	0	0	0	0	0	1	0	0	0	0	6.89
Study 3-11		1.56	1.25	0	40	10	0	0	0	0	0	0	0	0	0	0.1	0	0	0	0	0	7
Study 3-12		1.48	1.25	0	40	10	0	0	0	0	0	0	0	0	0	0.1	0	0	0	0	0	6.88
Study 3-13		1.68	1.25	0	40	10	0	0	0	0	0	0	0	0	0.1	0	0	0	0	0	0	7.03
Study 3-14		1.6	1.25	0	40	10	0	0	0	0	0	0	0	0	0.1	0	0	0	0	0	0	7.04
Study 3-15		1.6	1.25	0	40	10	0	0	0	0	0	0	0	0.005	0	0	0	0	0	0	0	7.02
Study 3-16		1.43	1.25	0	40	10	0	0	0	0	0	0	0	0.005	0	0	0	0	0	0	0	6.89
Study 3-17		1.75	1.25	0	40	10	0	0	0	0	0	0	0	0	0	0	0	0.5	0	0	0	7.04

Internal

Study 3-18		1.77	1.25	0	40	10	0	0	0	0	0	0	0	0	0	0	0	0.5	0	0	0	7.07
Study 3-19		1.71	1.25	0	40	10	0	0	0	0	0	0	0	0	0	0	0	0	0.1	0	0	7.1
Study 3-20		1.72	1.25	0	40	10	0	0	0	0	0	0	0	0	0	0	0	0	0.1	0	0	7.11
Study 3-21		1.39	1.25	0	40	10	0	0	15	0	0	0	0	0	0	0	0	0	0	0	0	7.09
Study 3-22		1.43	1.25	0	40	10	0	0	15	0	0	0	0	0	0	0	0	0	0	0	0	7.12
Study 4-1		0.47	1.56	0	40	10	160	0	0	0	0	0	100	0	0	0	0	0	0	0	0	7.2
Study 4-2		0.52	1.56	0	40	10	160	0	0	0	0	0	100	0	0	0	0	0	0	0	0	7.26
Study 5-1		0.51	0.85	0	40	10	160	0	0	0	0	0	100	0	0	0	0	0	0	0	0	7.72
Study 5-2		0.48	0.85	1	40	10	160	0	0	0	0	0	100	0	0	0	0	0	0	0	0	7.7
Study 6-1		0.56243503	0.082	0	0	10	160	0	0	0	0	0	0	0	0	0	0	0	0	10	50	5.45
Study 6-2		0.99701613	1.05	1	30	10	160	0	0	0	0	0	100	0	0	0	0	0	0	0	0	6.85
Study 7-1		0.48	4.7	0	30	10	160	0	0	0	0	0	100	0	0	0	0	0	0	0	0	7.57
Study 7-2		0.65	4.7	0	30	10	160	0	0	0	0	0	100	0	0	0	0	0	0	0	0	7.66
Study 8-1		0.48	2.67	0	30	10	160	0	0	0	0	0	100	0	0	0	0	0	0	0	0	6.64
Study 8-2		0.64	2.67	0	30	10	160	0	0	0	0	0	100	0	0	0	0	0	0	0	0	6.72
Study 8-3		0.9	0.267	0	30	10	160	0	0	0	0	0	100	0	0	0	0	0	0	0	0	5.71
Study 8-4		0.93	0.267	0	30	10	160	0	0	0	0	0	100	0	0	0	0	0	0	0	0	5.75
Study 9-1		0.63	0.78	0	30	10	160	0	0	0	0	0	100	0	0	0	0	0	0	0	0	6.96
Study 9-2		0.6	0.78	0	30	10	160	0	0	0	0	0	100	0	0	0	0	0	0	0	0	6.94
Study 9-3		0.6	0.78	0	30	10	160	5	0	0	0	0	100	0	0	0	0	0	0	0	0	6.93
Study 9-4		0.55	0.78	0	30	10	160	5	0	0	0	0	100	0	0	0	0	0	0	0	0	6.98
Study 9-5		0.7	0.78	0	30	10	160	10	0	0	0	0	100	0	0	0	0	0	0	0	0	7.03
Study 9-6		0.53	0.78	0	30	10	160	10	0	0	0	0	100	0	0	0	0	0	0	0	0	6.85
Study 9-7		0.79	0.78	0.5	30	10	160	0	0	0	0	0	100	0	0	0	0	0	0	0	0	7.06
Study 9-8		0.79	0.78	0.5	30	10	160	0	0	0	0	0	100	0	0	0	0	0	0	0	0	7.05
Study 9-9		0.73	0.78	0.5	30	10	160	5	0	0	0	0	100	0	0	0	0	0	0	0	0	7.03
Study 9-10		0.58	0.78	0.5	30	10	160	5	0	0	0	0	100	0	0	0	0	0	0	0	0	7.05
Study 10-1		1.26	1.05	1	15	10	160	0	0	0	0	0	100	0	0	0	0	0	0	0	0	6.34
Study 10-2		1.27	1.05	1	15	10	160	0	0	0	0	0	100	0	0	0	0	0	0	0	0	6.4
Study 10-3		1.26	1.05	1	15	10	160	0	0	0	0	0	100	0	0	0	0	0	0	0	0	6.34
Study 10-4		1.27	1.05	1	15	10	160	0	0	0	0	0	100	0	0	0	0	0	0	0	0	6.4
Study 11-1		1.68	1.25	0	40	10	0	0	0	0	0	0	0	0	0	0	0	0	0	0	0	6.87
Study 11-2		1.5	1.25	0	40	10	0	0	0	0	0	0	0	0	0	0	0	0	0	0	0	6.85
Study 11-3		1.5	1.25	1	40	10	0	0	0	0	0	0	0	0	0	0	0	0	0	0	0	6.85
Study 11-4		1.47	1.25	1	40	10	0	0	0	0	0	0	0	0	0	0	0	0	0	0	0	6.82
Study 11-5		1.85	1.25	5	40	10	0	0	0	0	0	0	0	0	0	0	0	0	0	0	0	7.04
Study 11-6		1.61	1.25	5	40	10	0	0	0	0	0	0	0	0	0	0	0	0	0	0	0	6.9
Study 11-7		0.9	1.25	10	40	10	0	0	0	0	0	0	0	0	0	0	0	0	0	0	0	7.03
Study 11-8		0.74	1.25	10	40	10	0	0	0	0	0	0	0	0	0	0	0	0	0	0	0	6.88
Study 11-9		0.91	1.25	5	40	10	160	0	0	0	0	0	0	0	0	0	0	0	0	0	0	7.06
Study 11-10		0.76	1.25	5	40	10	160	0	0	0	0	0	0	0	0	0	0	0	0	0	0	6.89
Study 11-11		0.99	1.25	10	40	10	160	0	0	0	0	0	0	0	0	0	0	0	0	0	0	7.07
Study 11-12		0.99	1.25	10	40	10	160	0	0	0	0	0	0	0	0	0	0	0	0	0	0	7.07
Study 11-13		1.02	1.25	5	40	10	160	0	10	0	0	0	0	0	0	0	0	0	0	0	0	7.07
Study 11-14		0.95	1.25	5	40	10	160	0	10	0	0	0	0	0	0	0	0	0	0	0	0	7.02
Study 11-15		0.75	1.25	0	40	10	160	0	0	0	0	0	0	0	0	0	0	0	0	0	0	7.02
Study 11-16		0.62	1.25	0	40	10	160	0	0	0	0	0	0	0	0	0	0	0	0	0	0	6.86
Study 12-1		0.67402184	0.185	0	30	10	160	0	0	0	0	0	100	0	0	0	0	0	0	0	0	6.96
Study 12-2		0.63793844	0.185	0	30	10	160	5	0	0	0	0	100	0	0	0	0	0	0	0	0	6.99
Study 12-3		1.079041	0.185	1	30	10	160	0	0	0	0	0	100	0	0	0	0	0	0	0	0	7.04
Study 13-1		0.83	0.185	0	15	10	160	0	0	0	0	0	100	0	0	0	0	0	0	0	0	6.25
Study 13-2		0.71	0.185	0	15	10	160	0	0	0	0	0	100	0	0	0	0	0	0	0	0	6.18
Study 13-3		0.99	0.185	0	15	10	160	0	0	0	0	0	100	0	0	0	0	0	0	0	0	6.25
Study 13-4		0.92	0.185	0	15	10	160	0	0	0	0	0	100	0	0	0	0	0	0	0	0	6.18
Study 13-5	Data added to step 2	0.92	0.185	0	15	10	160	0	0	0	0	0	100	0	0	0	0	0	0	0	0	6.18
Study 14-1	model generation /	1.03	1.25	0	45	15	15	5	5	0	0	0	40	0	0	0	0	0	0	0	0	7.15
Study 14-2	Dataset used for step	1.06	1.25	0	45	15	15	5	5	0	0	0	40	0	0	0	0	0	0	0	0	7.18

Study 14-3	3	0.87	0.63	0	40	10	230	2	0	0	0	0	120	0	0	0	0	0	0	0	0	6.15
Study 14-4		0.81	0.63	0	40	10	230	2	0	0	0	0	120	0	0	0	0	0	0	0	0	6.12
Study 14-5		1.04	1.25	0	45	15	65	5	3	0	0	0	0	0	0	0	0	0	0	0	0	7.16
Study 14-6		0.94	1.25	0	45	15	65	5	3	0	0	0	0	0	0	0	0	0	0	0	0	7.12
Study 14-7		0.52	0.51	0	30	10	180	7	0	0	0	0	115	0	0	0	0	0	0	0	0	7.73
Study 14-8		0.57	0.51	0	30	10	180	7	0	0	0	0	115	0	0	0	0	0	0	0	0	7.78
Study 14-9		1.13	1.25	6.5	45	10	10	5	1	0	0	0	25	0	0	0	0	0	0	0	0	7.14
Study 14-10		1.23	1.25	6.5	45	10	10	5	1	0	0	0	25	0	0	0	0	0	0	0	0	7.06
Study 14-11		1.75	0.037	3.5	35	10	180	0	0	0	0	0	120	0	0	0	0	0	0	0	0	5.57
Study 14-12		1.61	0.037	3.5	35	10	180	0	0	0	0	0	120	0	0	0	0	0	0	0	0	5.52
Study 14-13		2.44	0.037	15	15	50	250	30	30	0	0	0	200	0	0	0	0	0	0	0	0	5.7
Study 14-14		2.39	0.037	15	15	50	250	30	30	0	0	0	200	0	0	0	0	0	0	0	0	5.65
Study 14-15		2.39	0.37	15	15	50	250	30	30	0	0	0	200	0	0	0	0	0	0	0	0	6.65
Study 14-16		2.1	0.37	15	15	50	250	30	30	0	0	0	200	0	0	0	0	0	0	0	0	6.63
Study 14-17		2.16	0.12	8	15	50	250	30	30	0	0	0	200	0	0	0	0	0	0	0	0	6.21
Study 14-18		2.17	0.12	8	15	50	250	30	30	0	0	0	200	0	0	0	0	0	0	0	0	6.21
Study 14-19		2.37	0.12	13	15	50	250	30	30	0	0	0	200	0	0	0	0	0	0	0	0	6.2
Study 14-20		2.14	0.12	13	15	50	250	30	30	0	0	0	200	0	0	0	0	0	0	0	0	6.15
Study 15-1	Data added to step 4	0.76	1.2	0	40	10	160	0	0	0	0	0	0	0	0	0	0	0	0	0	0	8.16
Study 15-2	model generation	0.85	1.2	0	40	10	160	0	0	0	0	0	0	0	0	0	0	0	0	0	0	8.12
Study 15-3		0.81	1.2	0	40	10	160	0	0	0	0	0	0	0	0	0	0	0	0	0	0	7.36
Study 15-4		0.81	1.2	0	40	10	160	0	0	0	0	0	0	0	0	0	0	0	0	0	0	7.3
Study 15-5		0.93	1.2	0	40	10	160	0	0	0	0	0	0	0	0	0	0	0	0	0	0	6.17
Study 15-6		0.91	1.2	0	40	10	160	0	0	0	0	0	0	0	0	0	0	0	0	0	0	6.15
Study 15-7		1.21	1.2	0	40	10	160	0	0	0	0	0	0	0	0	0	0	0	0	0	0	5.08
Study 15-8		1.26	1.2	0	40	10	160	0	0	0	0	0	0	0	0	0	0	0	0	0	0	5.13
Study 15-9		0.71	1.2	0.5	40	10	160	0	0	0	0	0	0	0	0	0	0	0	0	0	0	7.25
Study 15-10		0.72	1.2	0.5	40	10	160	0	0	0	0	0	0	0	0	0	0	0	0	0	0	7.28
Study 15-11		0.85	1.2	2	40	10	160	0	0	0	0	0	0	0	0	0	0	0	0	0	0	7.32
Study 15-12		0.72	1.2	2	40	10	160	0	0	0	0	0	0	0	0	0	0	0	0	0	0	7.26
Study 15-13		0.85	1.2	5	40	10	160	0	0	0	0	0	0	0	0	0	0	0	0	0	0	7.3
Study 15-14		0.82	1.2	5	40	10	160	0	0	0	0	0	0	0	0	0	0	0	0	0	0	7.31
Study 15-15		0.68	1.2	0	40	10	160	0	5	0	0	0	0	0	0	0	0	0	0	0	0	7.25
Study 15-16		0.69	1.2	0	40	10	160	0	5	0	0	0	0	0	0	0	0	0	0	0	0	7.27
Study 15-17		0.96	1.3	0	30	10	160	0	0	0	0	0	100	0	0	0	0	0	0	0	0	7.2
Study 15-18		0.91	1.3	0	30	10	160	0	0	0	0	0	100	0	0	0	0	0	0	0	0	7.18
Study 16-1		1	1.2	0	30	10	180	7	0	0	0	0	115	0	0	0	0	0	0	0	0	7.79
Study 16-2		0.87	1.2	0	30	10	180	7	0	0	0	0	115	0	0	0	0	0	0	0	0	7.77
Study 16-3		0.99	1.3	0	30	10	180	7	0	0	0	0	115	0	0	0	0	0	0	0	0	7.75
Study 16-4		0.96	1.3	0	30	10	180	7	0	0	0	0	115	0	0	0	0	0	0	0	0	7.71
Study 16-5		1.08	1.17	0	30	10	180	7	0	0	0	0	115	0	0	0	0	0	0	0	0	6.96
Study 16-6		1.02	1.17	0	30	10	180	7	0	0	0	0	115	0	0	0	0	0	0	0	0	6.83
Study 16-7		1.1	1.17	0	30	10	180	7	0	0	0	0	115	0	0	0	0	0	0	0	0	7.22
Study 16-8		1.12	1.17	0	30	10	180	7	0	0	0	0	115	0	0	0	0	0	0	0	0	7.16
Study 16-9		0.57	1.2	0	30	10	180	7	0	0	0	0	0	0	0	0	0	0	0	0	0	7.81
Study 16-10		0.52	1.2	0	30	10	180	7	0	0	0	0	0	0	0	0	0	0	0	0	0	7.79
Study 17-1	Data added to step 5	1.2	0.12	0	40	10	160	5	0	0	0	0	0	0	0	0	0	0	0	0	0	7.16
Study 17-2	model generation	1.21	0.12	0	40	10	160	5	0	0	0	0	0	0	0	0	0	0	0	0	0	7.12
Study 17-3		1.38	0.12	0.5	40	10	160	0	0	0	0	0	0	0	0	0	0	0	0	0	0	7.18
Study 17-4		1.25	0.12	0.5	40	10	160	0	0	0	0	0	0	0	0	0	0	0	0	0	0	7.14
Study 17-5		1.29	0.12	0	40	10	160	0	0	0	0	0	0	0	0	0	0	0	0	0	0	7.15
Study 17-6		1.29	0.12	0	40	10	160	0	0	0	0	0	0	0	0	0	0	0	0	0	0	7.17
Study 18-1		1.1	0.12	0	40	10	160	0	15	0	0	0	0	0	0	0	0	0	0	0	0	6.99
Study 18-2		1.17	0.12	0	40	10	160	0	15	0	0	0	0	0	0	0	0	0	0	0	0	7.03
Study 18-3		1.04	0.12	0	40	10	160	0	0	0	0	0	100	0	0	0	0	0	0	0	0	6.97
Study 18-4		0.96	0.12	0	40	10	160	0	0	0	0	0	100	0	0	0	0	0	0	0	0	6.91
Study 19-1		0.39	1.5	0	40	10	160	0	0	0	0	0	100	0	0	0	0	0	0	0	0	7.05

Internal

Study 19-2		0.38	1.5	0	40	10	160	0	0	0	0	0	100	0	0	0	0	0	0	0	0	7.12
Study 20-1		0.68	1.5	0	40	10	160	0	0	0	0	0	100	0	0	0	0	0	0	0	0	7.56
Study 20-2		0.72	1.5	0	40	10	160	0	0	0	0	0	100	0	0	0	0	0	0	0	0	7.51
Study 21-1		0.85	0.24	0	40	10	160	0	0	0	0	0	100	0	0	0	0	0	0	0	0	6.84
Study 21-2		1.02	0.24	0	40	10	160	0	0	0	0	0	100	0	0	0	0	0	0	0	0	7.24
Study 22-1		0.53	1.1	0	40	10	160	0	0	0	0	0	100	0	0	0	0	0	0	0	0	7.85
Study 22-2		0.53	1.1	0	40	10	160	0	0	0	0	0	100	0	0	0	0	0	0	0	0	7.85
Study 23-1		0.56	0.19	0	40	10	160	0	0	0	0	0	100	0	0	0	0	0	0	0	0	7.91
Study 23-2		0.67	0.19	0	40	10	160	0	0	0	0	0	100	0	0	0	0	0	0	0	0	7.96
Study 23-3		0.36	0.19	0.05	40	10	160	0	0	0	0	0	100	0	0	0	0	0	0	0	0	7.83
Study 23-4		0.41	0.19	0.05	40	10	160	0	0	0	0	0	100	0	0	0	0	0	0	0	0	7.83
Study 23-5		0.4	0.19	1.4	40	10	160	0	0	0	0	0	100	0	0	0	0	0	0	0	0	7.94
Study 23-6		0.41	0.19	1.4	40	10	160	0	0	0	0	0	100	0	0	0	0	0	0	0	0	7.94
Study 23-7		1.08	0.001	0	40	10	160	0	0	0	0	0	100	0	0	0	0	0	0	0	0	5.67
Study 23-8		1.13	0.001	0	40	10	160	0	0	0	0	0	100	0	0	0	0	0	0	0	0	5.66
Study 23-9		0.86	0.001	0.05	40	10	160	0	0	0	0	0	100	0	0	0	0	0	0	0	0	5.8
Study 23-10		0.98	0.001	0.05	40	10	160	0	0	0	0	0	100	0	0	0	0	0	0	0	0	5.73
Study 23-11		1.3	0.001	1.4	40	10	160	0	0	0	0	0	100	0	0	0	0	0	0	0	0	5.87
Study 23-12		1.25	0.001	1.4	40	10	160	0	0	0	0	0	100	0	0	0	0	0	0	0	0	5.77
Study 24-1		0.41	0.29	0	40	10	160	0	0	0	0	0	100	0	0	0	0	0	0	0	0	7.6
Study 24-2		0.34	0.29	0	40	10	160	0	0	0	0	0	100	0	0	0	0	0	0	0	0	7.6
Study 24-3		0.46	0.29	0.05	40	10	160	0	0	0	0	0	100	0	0	0	0	0	0	0	0	7.63
Study 24-4		0.35	0.29	0.05	40	10	160	0	0	0	0	0	100	0	0	0	0	0	0	0	0	7.58
Study 24-5		0.43	0.29	1.4	40	10	160	0	0	0	0	0	100	0	0	0	0	0	0	0	0	7.57
Study 24-6		0.41	0.29	1.4	40	10	160	0	0	0	0	0	100	0	0	0	0	0	0	0	0	7.57
Study 24-7		0.35	0.29	0.05	40	10	160	0	0	0	0	0	100	0	0	0	0	0	0	0	0	7.58
Study 24-8		0.41	0.29	0.05	40	10	160	0	0	0	0	0	100	0	0	0	0	0	0	0	0	7.61
Study 24-9		0.38	0.29	1.4	40	10	160	0	0	0	0	0	100	0	0	0	0	0	0	0	0	7.65
Study 24-10		0.37	0.29	1.4	40	10	160	0	0	0	0	0	100	0	0	0	0	0	0	0	0	7.63
Study 25-1		0.61	0.21	0	40	10	160	0	0	0	0	0	100	0	0	0	0	0	0	0	0	7.91
Study 25-2		0.62	0.21	0	40	10	160	0	0	0	0	0	100	0	0	0	0	0	0	0	0	7.94
Study 26-1		0.45	0.29	0	40	10	160	0	0	0	0	0	100	0	0	0	0	0	0	0	0	7.66
Study 26-2		0.4	0.29	0	40	10	160	0	0	0	0	0	100	0	0	0	0	0	0	0	0	7.61
Study 26-3		0.46	0.29	0	40	20	160	0	0	0	0	0	100	0	0	0	0	0	0	0	0	7.65
Study 26-4		0.43	0.29	0	40	20	160	0	0	0	0	0	100	0	0	0	0	0	0	0	0	7.62
