# Supplementary material for: Bayesian optimization and machine learning for vaccine formulation development
Source: PLoS One. 2025 Jun 11;20(6):e0324205. doi: 10.1371/journal.pone.0324205 (PMC12157168; doi:10.1371/journal.pone.0324205)
Supplement: S2 Table — True experimental value of infectious titer loss for each study were compared against ML predicted values for infectious titer loss (log10 PFU/mL) using model generated in step 3–5. The test dataset is comprised of 10% data points used in the generation of the model. (PDF) [file pone.0324205.s003.pdf]

S2 Table. Residual rHSA and spiked rHSA values for the test dataset in case 1. True experimental value of infectious titer loss for each study were compared against ML predicted values for infectious titer loss (log_{10} PFU/mL) using model generated in step 3 to 5. The test dataset is comprised of 10% data points used in the generation of the model

	**Study ID**	**Residual rHSA concentration (mg/mL)**	**Spiked rHSA concentration (mg/mL)**	**True Value**		**ML Predicted value In Step 3**		**ML Predicted value In Step 4**		**ML Predicted value In Step 5**	
				**Average Titer Loss, log10pfu/mL**	**95% CI**	**Average Titer Loss, log10pfu/mL**	**95% CI**	**Average Titer Loss, log10pfu/mL**	**95% CI**	**Average Titer Loss, log10pfu/mL**	**95% CI**
**Test Set**	CA-21-028 F13-1	1.250	0.000	1.390	0.07	1.400	0.02	1.360	0.08	1.350	0.08
	CA-21-054 F6-1	1.250	0.000	1.560	0.07	1.440	0.07	1.480	0.06	1.490	0.05
	CA-21-054 F5-1	1.250	0.000	1.420	0.07	1.390	0.04	1.360	0.09	1.350	0.10
	CA-21-085 (F1-1)	1.250	0.000	1.680	0.07	1.630	0.10	1.640	0.09	1.630	0.11
	CA-21-085 (F6-1)	1.250	5.000	0.910	0.06	0.910	0.03	0.890	0.03	0.870	0.06
	CA-22-058 F1-1	0.190	0.000	0.560	0.02	0.550	0.03	0.560	0.03	0.570	0.03
	CA-22-058 F6-2	0.001	0.000	1.130	0.07	0.880	0.07	0.960	0.13	1.090	0.04
	CA-21-028 F2-1	1.250	0.000	1.380	0.05	1.380	0.02	1.410	0.06	1.380	0.02
	CA-21-028 F7-1	1.250	0.000	0.660	0.06	0.680	0.07	0.700	0.08	0.700	0.06
	CA-21-028 F11-1	1.250	0.000	1.020	0.04	1.100	0.18	1.040	0.01	1.110	0.18
	CA-21-108 (F1-1)	1.250	0.000	1.030	0.02	1.040	0.01	1.040	0.01	1.020	0.03
	CA-21-108 (F2-1)	0.630	0.000	0.870	0.04	0.840	0.04	0.880	0.07	0.860	0.02
	CA-21-108 (F5-1)	1.250	6.500	1.130	0.09	1.140	0.03	1.140	0.03	1.160	0.05
	CA-21-106 (F5-1)	1.200	0.500	0.710	0.06			0.710	0.00	0.730	0.03
	CA-21-106 (F6-1)	1.200	2.000	0.850	0.05			0.840	0.02	0.830	0.03
	CA-21-126 (F5-1)	1.200	0.000	0.570	0.03			0.610	0.13	0.560	0.02
	CA-21-146 (F1-1)	0.120	0.000	1.200	0.15					1.220	0.03
	CA-22-058 F7-2	0.001	0.050	0.980	0.08					0.980	0.04
	CA-22-058 F8-2	0.001	1.400	1.250	0.06					1.220	0.09
	CA-22-058 F2-2	0.190	0.050	0.410	0.06					0.410	0.05
