# Supplementary material for: Bayesian optimization and machine learning for vaccine formulation development
Source: PLoS One. 2025 Jun 11;20(6):e0324205. doi: 10.1371/journal.pone.0324205 (PMC12157168; doi:10.1371/journal.pone.0324205)
Supplement: S3 Table — True experimental value of infectious titer loss for each study were compared against ML predicted values for infectious titer loss (log10 PFU/mL) using model generated in step 3–5. The holdout dataset is comprised of 22 data points not used in the generation of the model (i.e., unforeseen data). (PDF) [file pone.0324205.s004.pdf]

S3 Table. Residual rHSA and spiked rHSA values for the holdout dataset in case 1. True experimental value of infectious titer loss for each study were compared against ML predicted values for infectious titer loss (log_{10} PFU/mL) using model generated in step 3 to 5. The holdout dataset is comprised of 22 data points not used in the generation of the model (i.e. unforeseen data).

	Study ID	Residual rHSA concentration (mg/mL)	Spiked rHSA concentration (mg/mL)	True Value		ML Predicted value In Step 3		ML Predicted value In Step 4		ML Predicted value In Step 5	
				Average Titer Loss, log10pfu/mL	95% CI	Average Titer Loss, log10pfu/mL	95% CI	Average Titer Loss, log10pfu/mL	95% CI	Average Titer Loss, log10pfu/mL	95% CI
**Holdout set**	CA-22-125	0.291	0.000	0.450	0.06	0.550	0.03	0.550	0.03	0.430	0.02
	CA-22-148	0.086	0.000	0.470	0.06	0.550	0.04	0.560	0.04	0.500	0.02
	CA-22-149	0.023	0.000	0.650	0.05	0.570	0.03	0.570	0.03	0.480	0.02
	CA-23-003	0.075	0.000	0.560	0.05	0.570	0.04	0.570	0.04	0.480	0.03
	CA-23-041-F1	0.022	0.000	0.660	0.09	0.560	0.03	0.580	0.04	0.620	0.06
	CA-23-041-F2	0.022	0.078	0.640	0.04	0.580	0.01	0.590	0.04	0.590	0.07
	CA-23-041-F3	0.022	0.278	0.660	0.06	0.620	0.05	0.610	0.05	0.550	0.06
	CA-23-041-F4	0.022	0.478	0.720	0.05	0.650	0.08	0.630	0.06	0.580	0.07
	CA-23-041-F5	0.022	0.978	0.720	0.03	0.760	0.15	0.700	0.15	0.590	0.07
	CA-23-041-F6	0.022	2.978	0.770	0.09	0.920	0.18	0.890	0.16	0.650	0.13
	CA-23-041-F7	0.022	4.978	0.780	0.01	0.970	0.17	0.950	0.17	0.670	0.13
	CA-23-067 F1	0.021	0.000	0.835	0.08	0.580	0.03	0.610	0.04	0.890	0.09
	CA-23-067 F2-10	0.021	0.000	0.795	0.06	0.580	0.03	0.610	0.04	0.810	0.09
	CA-23-067 F2-25	0.021	0.000	0.835	0.09	0.630	0.04	0.720	0.10	0.860	0.12
	CA-23-068 F1	0.000	0.000	0.790	0.09	0.590	0.03	0.610	0.03	0.950	0.09
	CA-23-068 F2	0.000	0.010	0.820	0.10	0.580	0.03	0.610	0.04	0.890	0.08
	CA-23-068 F3	0.000	0.050	0.805	0.11	0.590	0.03	0.620	0.05	0.790	0.09
	CA-23-068 F4	0.000	0.100	0.835	0.13	0.600	0.01	0.620	0.05	0.710	0.10
	CA-23-068 F5	0.000	0.500	0.775	0.10	0.690	0.07	0.670	0.07	0.780	0.08
	CA-23-068 F6	0.000	1.000	0.730	0.05	0.840	0.13	0.750	0.13	0.800	0.08
	CA-23-068 F7	0.000	1.500	0.685	0.10	0.880	0.13	0.790	0.12	0.840	0.08
	CA-23-068 F8	0.000	3.000	0.705	0.07	0.980	0.15	0.930	0.15	0.870	0.12
