# Supplementary material for: Bayesian optimization and machine learning for vaccine formulation development
Source: PLoS One. 2025 Jun 11;20(6):e0324205. doi: 10.1371/journal.pone.0324205 (PMC12157168; doi:10.1371/journal.pone.0324205)
Supplement: S4 Table — (PDF) [file pone.0324205.s005.pdf]

S4 Table. Data used for generating Fig 7 heatmap prediction

Spiked rHSA (mg/mL)	Residual rHSA (mg/mL)	Starting Titre (Logpfu/mL)	Predicted infectious titer loss, (log pfu/mL)	Confidence interval for predicted titer loss
0	0	7.6	0.45	0.03
0	0.2	7.6	0.42	0.03
0	0.4	7.6	0.41	0.03
0	0.6	7.6	0.44	0.03
0	0.8	7.6	0.49	0.04
0	1	7.6	0.51	0.04
0	1.2	7.6	0.56	0.04
0	1.4	7.6	0.59	0.05
0	1.6	7.6	0.6	0.05
0	1.8	7.6	0.6	0.05
0	2	7.6	0.6	0.05
0	2.2	7.6	0.6	0.05
0	2.4	7.6	0.6	0.05
0	2.6	7.6	0.6	0.05
0	2.8	7.6	0.6	0.05
0	3	7.6	0.61	0.05
0.2	0	7.6	0.43	0.04
0.2	0.2	7.6	0.4	0.03
0.2	0.4	7.6	0.4	0.02
0.2	0.6	7.6	0.43	0.02
0.2	0.8	7.6	0.47	0.02
0.2	1	7.6	0.49	0.02
0.2	1.2	7.6	0.52	0.03
0.2	1.4	7.6	0.54	0.03
0.2	1.6	7.6	0.55	0.04
0.2	1.8	7.6	0.55	0.04
0.2	2	7.6	0.56	0.04
0.2	2.2	7.6	0.56	0.04
0.2	2.4	7.6	0.56	0.04
0.2	2.6	7.6	0.56	0.04
0.2	2.8	7.6	0.56	0.04
0.2	3	7.6	0.57	0.04
0.4	0	7.6	0.44	0.04
0.4	0.2	7.6	0.41	0.03
0.4	0.4	7.6	0.41	0.02
0.4	0.6	7.6	0.44	0.02
0.4	0.8	7.6	0.47	0.02
0.4	1	7.6	0.49	0.03
0.4	1.2	7.6	0.52	0.03
0.4	1.4	7.6	0.54	0.03
0.4	1.6	7.6	0.55	0.04
0.4	1.8	7.6	0.56	0.04
0.4	2	7.6	0.56	0.04
0.4	2.2	7.6	0.56	0.04
0.4	2.4	7.6	0.56	0.04

0.4	2.6	7.6	0.56	0.04
0.4	2.8	7.6	0.56	0.04
0.4	3	7.6	0.57	0.04
0.6	0	7.6	0.44	0.04
0.6	0.2	7.6	0.41	0.03
0.6	0.4	7.6	0.41	0.02
0.6	0.6	7.6	0.44	0.02
0.6	0.8	7.6	0.47	0.02
0.6	1	7.6	0.49	0.03
0.6	1.2	7.6	0.53	0.03
0.6	1.4	7.6	0.55	0.04
0.6	1.6	7.6	0.55	0.04
0.6	1.8	7.6	0.56	0.04
0.6	2	7.6	0.56	0.04
0.6	2.2	7.6	0.56	0.04
0.6	2.4	7.6	0.56	0.04
0.6	2.6	7.6	0.56	0.04
0.6	2.8	7.6	0.57	0.04
0.6	3	7.6	0.57	0.04
0.8	0	7.6	0.45	0.04
0.8	0.2	7.6	0.42	0.03
0.8	0.4	7.6	0.42	0.02
0.8	0.6	7.6	0.45	0.02
0.8	0.8	7.6	0.48	0.02
0.8	1	7.6	0.5	0.02
0.8	1.2	7.6	0.53	0.03
0.8	1.4	7.6	0.55	0.03
0.8	1.6	7.6	0.56	0.03
0.8	1.8	7.6	0.56	0.04
0.8	2	7.6	0.56	0.03
0.8	2.2	7.6	0.56	0.03
0.8	2.4	7.6	0.56	0.03
0.8	2.6	7.6	0.56	0.03
0.8	2.8	7.6	0.57	0.04
0.8	3	7.6	0.57	0.04
1	0	7.6	0.46	0.04
1	0.2	7.6	0.42	0.03
1	0.4	7.6	0.42	0.02
1	0.6	7.6	0.45	0.02
1	0.8	7.6	0.48	0.02
1	1	7.6	0.5	0.02
1	1.2	7.6	0.53	0.03
1	1.4	7.6	0.55	0.03
1	1.6	7.6	0.56	0.03
1	1.8	7.6	0.56	0.03
1	2	7.6	0.56	0.03
1	2.2	7.6	0.56	0.03
1	2.4	7.6	0.56	0.03

1	2.6	7.6	0.56	0.03
1	2.8	7.6	0.57	0.04
1	3	7.6	0.57	0.03
1.2	0	7.6	0.45	0.03
1.2	0.2	7.6	0.42	0.02
1.2	0.4	7.6	0.42	0.01
1.2	0.6	7.6	0.45	0.01
1.2	0.8	7.6	0.47	0.02
1.2	1	7.6	0.49	0.02
1.2	1.2	7.6	0.52	0.03
1.2	1.4	7.6	0.54	0.03
1.2	1.6	7.6	0.55	0.03
1.2	1.8	7.6	0.55	0.04
1.2	2	7.6	0.55	0.03
1.2	2.2	7.6	0.56	0.03
1.2	2.4	7.6	0.56	0.03
1.2	2.6	7.6	0.56	0.03
1.2	2.8	7.6	0.56	0.04
1.2	3	7.6	0.57	0.04
1.4	0	7.6	0.44	0.03
1.4	0.2	7.6	0.41	0.02
1.4	0.4	7.6	0.42	0.01
1.4	0.6	7.6	0.45	0.01
1.4	0.8	7.6	0.47	0.02
1.4	1	7.6	0.49	0.03
1.4	1.2	7.6	0.51	0.03
1.4	1.4	7.6	0.53	0.03
1.4	1.6	7.6	0.54	0.04
1.4	1.8	7.6	0.54	0.04
1.4	2	7.6	0.54	0.04
1.4	2.2	7.6	0.55	0.04
1.4	2.4	7.6	0.55	0.04
1.4	2.6	7.6	0.55	0.04
1.4	2.8	7.6	0.55	0.04
1.4	3	7.6	0.55	0.04
1.6	0	7.6	0.45	0.04
1.6	0.2	7.6	0.42	0.04
1.6	0.4	7.6	0.43	0.02
1.6	0.6	7.6	0.46	0.02
1.6	0.8	7.6	0.48	0.02
1.6	1	7.6	0.5	0.03
1.6	1.2	7.6	0.52	0.03
1.6	1.4	7.6	0.54	0.04
1.6	1.6	7.6	0.55	0.04
1.6	1.8	7.6	0.55	0.04
1.6	2	7.6	0.55	0.04
1.6	2.2	7.6	0.55	0.04
1.6	2.4	7.6	0.55	0.04

1.6	2.6	7.6	0.56	0.04
1.6	2.8	7.6	0.56	0.04
1.6	3	7.6	0.56	0.04
1.8	0	7.6	0.46	0.05
1.8	0.2	7.6	0.43	0.05
1.8	0.4	7.6	0.44	0.04
1.8	0.6	7.6	0.46	0.03
1.8	0.8	7.6	0.48	0.03
1.8	1	7.6	0.5	0.03
1.8	1.2	7.6	0.52	0.04
1.8	1.4	7.6	0.54	0.04
1.8	1.6	7.6	0.55	0.04
1.8	1.8	7.6	0.55	0.04
1.8	2	7.6	0.56	0.04
1.8	2.2	7.6	0.56	0.04
1.8	2.4	7.6	0.56	0.04
1.8	2.6	7.6	0.56	0.04
1.8	2.8	7.6	0.56	0.04
1.8	3	7.6	0.56	0.05
2	0	7.6	0.46	0.05
2	0.2	7.6	0.43	0.05
2	0.4	7.6	0.44	0.04
2	0.6	7.6	0.46	0.03
2	0.8	7.6	0.48	0.03
2	1	7.6	0.5	0.03
2	1.2	7.6	0.52	0.04
2	1.4	7.6	0.54	0.04
2	1.6	7.6	0.55	0.04
2	1.8	7.6	0.55	0.04
2	2	7.6	0.56	0.04
2	2.2	7.6	0.56	0.04
2	2.4	7.6	0.56	0.04
2	2.6	7.6	0.56	0.04
2	2.8	7.6	0.56	0.04
2	3	7.6	0.56	0.05
2.2	0	7.6	0.47	0.06
2.2	0.2	7.6	0.45	0.07
2.2	0.4	7.6	0.45	0.06
2.2	0.6	7.6	0.48	0.06
2.2	0.8	7.6	0.5	0.06
2.2	1	7.6	0.52	0.06
2.2	1.2	7.6	0.54	0.06
2.2	1.4	7.6	0.56	0.06
2.2	1.6	7.6	0.56	0.06
2.2	1.8	7.6	0.57	0.06
2.2	2	7.6	0.57	0.06
2.2	2.2	7.6	0.57	0.06
2.2	2.4	7.6	0.57	0.06

2.2	2.6	7.6	0.57	0.06
2.2	2.8	7.6	0.58	0.06
2.2	3	7.6	0.58	0.06
2.4	0	7.6	0.49	0.07
2.4	0.2	7.6	0.46	0.08
2.4	0.4	7.6	0.47	0.08
2.4	0.6	7.6	0.49	0.07
2.4	0.8	7.6	0.51	0.07
2.4	1	7.6	0.53	0.07
2.4	1.2	7.6	0.55	0.07
2.4	1.4	7.6	0.57	0.06
2.4	1.6	7.6	0.57	0.06
2.4	1.8	7.6	0.58	0.06
2.4	2	7.6	0.58	0.06
2.4	2.2	7.6	0.58	0.06
2.4	2.4	7.6	0.58	0.06
2.4	2.6	7.6	0.58	0.06
2.4	2.8	7.6	0.59	0.06
2.4	3	7.6	0.59	0.07
2.6	0	7.6	0.51	0.1
2.6	0.2	7.6	0.48	0.1
2.6	0.4	7.6	0.49	0.1
2.6	0.6	7.6	0.51	0.09
2.6	0.8	7.6	0.53	0.09
2.6	1	7.6	0.54	0.09
2.6	1.2	7.6	0.56	0.08
2.6	1.4	7.6	0.58	0.08
2.6	1.6	7.6	0.59	0.08
2.6	1.8	7.6	0.59	0.08
2.6	2	7.6	0.6	0.08
2.6	2.2	7.6	0.6	0.08
2.6	2.4	7.6	0.6	0.08
2.6	2.6	7.6	0.6	0.08
2.6	2.8	7.6	0.6	0.08
2.6	3	7.6	0.61	0.08
2.8	0	7.6	0.51	0.1
2.8	0.2	7.6	0.49	0.11
2.8	0.4	7.6	0.49	0.1
2.8	0.6	7.6	0.52	0.09
2.8	0.8	7.6	0.53	0.09
2.8	1	7.6	0.55	0.09
2.8	1.2	7.6	0.57	0.08
2.8	1.4	7.6	0.59	0.08
2.8	1.6	7.6	0.59	0.08
2.8	1.8	7.6	0.6	0.08
2.8	2	7.6	0.6	0.08
2.8	2.2	7.6	0.6	0.08
2.8	2.4	7.6	0.6	0.08

2.8	2.6	7.6	0.6	0.08
2.8	2.8	7.6	0.61	0.08
2.8	3	7.6	0.61	0.09
3	0	7.6	0.52	0.1
3	0.2	7.6	0.49	0.1
3	0.4	7.6	0.5	0.1
3	0.6	7.6	0.52	0.09
3	0.8	7.6	0.54	0.08
3	1	7.6	0.56	0.08
3	1.2	7.6	0.57	0.08
3	1.4	7.6	0.59	0.08
3	1.6	7.6	0.6	0.08
3	1.8	7.6	0.6	0.08
3	2	7.6	0.61	0.08
3	2.2	7.6	0.61	0.08
3	2.4	7.6	0.61	0.08
3	2.6	7.6	0.61	0.08
3	2.8	7.6	0.61	0.08
3	3	7.6	0.62	0.08
3.2	0	7.6	0.53	0.09
3.2	0.2	7.6	0.5	0.1
3.2	0.4	7.6	0.51	0.09
3.2	0.6	7.6	0.54	0.09
3.2	0.8	7.6	0.55	0.08
3.2	1	7.6	0.57	0.08
3.2	1.2	7.6	0.58	0.08
3.2	1.4	7.6	0.6	0.07
3.2	1.6	7.6	0.61	0.07
3.2	1.8	7.6	0.61	0.07
3.2	2	7.6	0.62	0.07
3.2	2.2	7.6	0.62	0.07
3.2	2.4	7.6	0.62	0.07
3.2	2.6	7.6	0.62	0.07
3.2	2.8	7.6	0.62	0.07
3.2	3	7.6	0.63	0.08
3.4	0	7.6	0.54	0.1
3.4	0.2	7.6	0.51	0.11
3.4	0.4	7.6	0.51	0.09
3.4	0.6	7.6	0.54	0.09
3.4	0.8	7.6	0.56	0.08
3.4	1	7.6	0.57	0.08
3.4	1.2	7.6	0.59	0.08
3.4	1.4	7.6	0.61	0.08
3.4	1.6	7.6	0.61	0.08
3.4	1.8	7.6	0.62	0.08
3.4	2	7.6	0.62	0.08
3.4	2.2	7.6	0.62	0.08
3.4	2.4	7.6	0.62	0.08

3.4	2.6	7.6	0.62	0.08
3.4	2.8	7.6	0.63	0.08
3.4	3	7.6	0.63	0.08
3.6	0	7.6	0.54	0.1
3.6	0.2	7.6	0.51	0.11
3.6	0.4	7.6	0.51	0.09
3.6	0.6	7.6	0.54	0.09
3.6	0.8	7.6	0.56	0.08
3.6	1	7.6	0.57	0.08
3.6	1.2	7.6	0.59	0.08
3.6	1.4	7.6	0.61	0.08
3.6	1.6	7.6	0.61	0.08
3.6	1.8	7.6	0.62	0.08
3.6	2	7.6	0.62	0.08
3.6	2.2	7.6	0.62	0.08
3.6	2.4	7.6	0.62	0.08
3.6	2.6	7.6	0.62	0.08
3.6	2.8	7.6	0.63	0.08
3.6	3	7.6	0.63	0.08
3.8	0	7.6	0.54	0.1
3.8	0.2	7.6	0.51	0.11
3.8	0.4	7.6	0.51	0.09
3.8	0.6	7.6	0.54	0.09
3.8	0.8	7.6	0.56	0.08
3.8	1	7.6	0.57	0.08
3.8	1.2	7.6	0.59	0.08
3.8	1.4	7.6	0.61	0.08
3.8	1.6	7.6	0.61	0.08
3.8	1.8	7.6	0.62	0.08
3.8	2	7.6	0.62	0.08
3.8	2.2	7.6	0.62	0.08
3.8	2.4	7.6	0.62	0.08
3.8	2.6	7.6	0.62	0.08
3.8	2.8	7.6	0.63	0.08
3.8	3	7.6	0.63	0.08
4	0	7.6	0.54	0.1
4	0.2	7.6	0.51	0.11
4	0.4	7.6	0.51	0.09
4	0.6	7.6	0.54	0.09
4	0.8	7.6	0.56	0.08
4	1	7.6	0.57	0.08
4	1.2	7.6	0.59	0.08
4	1.4	7.6	0.61	0.08
4	1.6	7.6	0.61	0.08
4	1.8	7.6	0.62	0.08
4	2	7.6	0.62	0.08
4	2.2	7.6	0.62	0.08
4	2.4	7.6	0.62	0.08

4	2.6	7.6	0.62	0.08
4	2.8	7.6	0.63	0.08
4	3	7.6	0.63	0.08
4.2	0	7.6	0.53	0.1
4.2	0.2	7.6	0.51	0.11
4.2	0.4	7.6	0.51	0.1
4.2	0.6	7.6	0.54	0.09
4.2	0.8	7.6	0.55	0.08
4.2	1	7.6	0.57	0.08
4.2	1.2	7.6	0.59	0.08
4.2	1.4	7.6	0.6	0.08
4.2	1.6	7.6	0.61	0.08
4.2	1.8	7.6	0.61	0.08
4.2	2	7.6	0.62	0.08
4.2	2.2	7.6	0.62	0.08
4.2	2.4	7.6	0.62	0.08
4.2	2.6	7.6	0.62	0.08
4.2	2.8	7.6	0.62	0.08
4.2	3	7.6	0.63	0.08
4.4	0	7.6	0.53	0.1
4.4	0.2	7.6	0.51	0.11
4.4	0.4	7.6	0.51	0.1
4.4	0.6	7.6	0.54	0.09
4.4	0.8	7.6	0.55	0.08
4.4	1	7.6	0.57	0.08
4.4	1.2	7.6	0.59	0.08
4.4	1.4	7.6	0.6	0.08
4.4	1.6	7.6	0.61	0.08
4.4	1.8	7.6	0.61	0.08
4.4	2	7.6	0.62	0.08
4.4	2.2	7.6	0.62	0.08
4.4	2.4	7.6	0.62	0.08
4.4	2.6	7.6	0.62	0.08
4.4	2.8	7.6	0.62	0.08
4.4	3	7.6	0.63	0.08
4.6	0	7.6	0.53	0.1
4.6	0.2	7.6	0.51	0.11
4.6	0.4	7.6	0.51	0.1
4.6	0.6	7.6	0.54	0.09
4.6	0.8	7.6	0.55	0.08
4.6	1	7.6	0.57	0.08
4.6	1.2	7.6	0.59	0.08
4.6	1.4	7.6	0.6	0.08
4.6	1.6	7.6	0.61	0.08
4.6	1.8	7.6	0.61	0.08
4.6	2	7.6	0.62	0.08
4.6	2.2	7.6	0.62	0.08
4.6	2.4	7.6	0.62	0.08

4.6	2.6	7.6	0.62	0.08
4.6	2.8	7.6	0.62	0.08
4.6	3	7.6	0.63	0.08
4.8	0	7.6	0.53	0.1
4.8	0.2	7.6	0.51	0.11
4.8	0.4	7.6	0.51	0.1
4.8	0.6	7.6	0.54	0.09
4.8	0.8	7.6	0.55	0.08
4.8	1	7.6	0.57	0.08
4.8	1.2	7.6	0.59	0.08
4.8	1.4	7.6	0.6	0.08
4.8	1.6	7.6	0.61	0.08
4.8	1.8	7.6	0.61	0.08
4.8	2	7.6	0.62	0.08
4.8	2.2	7.6	0.62	0.08
4.8	2.4	7.6	0.62	0.08
4.8	2.6	7.6	0.62	0.08
4.8	2.8	7.6	0.62	0.08
4.8	3	7.6	0.63	0.08
5	0	7.6	0.53	0.1
5	0.2	7.6	0.51	0.11
5	0.4	7.6	0.51	0.1
5	0.6	7.6	0.54	0.09
5	0.8	7.6	0.55	0.08
5	1	7.6	0.57	0.08
5	1.2	7.6	0.59	0.08
5	1.4	7.6	0.6	0.08
5	1.6	7.6	0.61	0.08
5	1.8	7.6	0.61	0.08
5	2	7.6	0.62	0.08
5	2.2	7.6	0.62	0.08
5	2.4	7.6	0.62	0.08
5	2.6	7.6	0.62	0.08
5	2.8	7.6	0.62	0.08
5	3	7.6	0.63	0.08
