# Supplementary material for: Bayesian optimization and machine learning for vaccine formulation development
Source: PLoS One. 2025 Jun 11;20(6):e0324205. doi: 10.1371/journal.pone.0324205 (PMC12157168; doi:10.1371/journal.pone.0324205)
Supplement: S5 Table — (PDF) [file pone.0324205.s006.pdf]

S5 Table. Data used for case 2 model generation

P	G (g/L)	PVP10 (g/L	K (g/L)	Proline (g/	M (g/L)	Sorbitol (g/	J (g/L)	L (g/L)	Tg' (°C)
2.423	100	20	7	20	0	0	2.5	0.05	-35
2.423	100	20	1	20	0	0	5	0.05	-34
2.423	100	10	10	1	0.3	5	10	0.01	-37
2.423	100	20	0	20	0	0	5	0.05	-34
2.423	150	20	1.325	3.2	0.337	30	2.5	5	-33
2.423	100	10	10	30	0.3	55	10	0.01	-39
2.423	100	50	10	30	0.3	5	10	0.01	-37
2.423	100	7	1	20	0	0	5	0.05	-35
2.423	100	10	1	30	0.337	5	10	0.01	-38
2.423	100	30	1	20	0.337	0	5	0.05	-32
2.423	100	20	1.3	20	0	0	5	0.5	-34
2.423	100	10	1	1	0.337	5	10	5	-34
2.423	100	30	1	20	0.337	0	10	0.01	-35
2.423	200	20	7	20	0	0	5	0.05	-33
2.423	150	0	0	3.2	0.337	30	2.5	5	-37
2.423	100	30	1	20	0.337	0	10	0.01	-34
2.423	100	20	1.3	20	0	0	5	0.05	-34
2.423	100	20	1.3	20	0	0	5	0.05	-34
2.423	100	20	1.3	20	0	0	5	0	-34
2.423	100	10	10	30	0.3	55	1	5	-38
2.423	100	20	0.5	20	0	0	5	0.05	-34.5
2.423	110	0	0	5.1	0	0	5.5	1.05	-34
2.423	140	0	0	10	0	0	10	2.05	-35
2.423	110	0	0	5.1	0	0	5.5	1.1	-35
2.423	100	50	1	30	0.3	5	10	5	-36
2.423	100	30	5.5	30	0.3	30	0	0	-36
2.423	100	50	10	1	0.337	5	10	5	-35
2.423	100	50	10	1	0.337	5	10	0.01	-34
2.423	100	30	1	20	0	0	20	0.01	-37
2.423	150	20	1.325	3.2	0.337	30	2.5	0	-32
2.423	110	0	0	5.1	0	0	5.5	1.05	-35
2.423	100	10	1	30	0.3	5	10	0.01	-38
2.423	150	20	1.325	3.2	0	30	2.5	5	-33
2.423	100	20	0	40	0	0	5	0.05	-35
2.423	140	0	0	0.2	0	0	10	2.05	-34
2.423	80	0	0	10	0	0	10	2.1	-39
2.423	150	20	1.325	3.2	0.337	30	2.5	5	-33
2.423	100	0	1	20	0	0	5	0.05	-39
2.423	140	0	0	0.2	0	0	10	1.05	-34
2.423	140	0	0	10	0	0	10	0.05	-35
2.423	150	20	1.325	3.2	0.337	30	2.5	0.05	-33
2.423	150	0	1.325	3.2	0	30	2.5	5	-36
2.423	100	10	10	30	0.3	5	1	0.01	-37
2.423	100	30	1	40	0.337	0	5	0.01	-36
2.423	150	20	1.325	3.2	0.337	30	2.5	5	-30
2.423	100	13	0.883	2.133	0.225	20	1.667	3.333	-34
2.423	150	20	1.325	3.2	0.337	30	2.5	5	-33
2.423	100	50	1	30	0.3	5	1	0.01	-35
2.423	100	10	10	1	0.337	5	10	0.01	-36

2.423	100	50	1	1	0.337	5	10	5	-32
2.423	150	20	1.325	3.2	0.337	30	2.5	0.05	-32
2.423	100	20	10	1	0.337	5	5	0.05	-34
2.423	75	10	0.663	1.6	0.196	15	1.25	2.5	-35
2.423	140	0	0	10	0	0	1	2.05	-34
2.423	150	20	1.325	0	0	30	2.5	5	-33
2.423	100	10	10	30	0.3	5	10	5	-39
2.423	150	20	0	0	0	30	2.5	5	-33
2.423	150	20	1.325	3.2	0	30	0	5	-32
2.423	100	10	1	30	0.3	5	1	5	-37
2.423	150	0	1.325	3.2	0.337	0	2.5	5	-34
2.423	100	10	1	30	0.3	55	1	0.01	-38
2.423	150	20	1.325	3.2	0.337	30	2.5	5	-33
2.423	150	20	1.325	3.2	0.337	30	2.5	0.5	-34
2.423	100	50	10	30	0.3	5	1	5	-36
2.423	100	30	5.5	30	0.3	30	0	0	-37
2.423	100	50	1	1	0.337	5	10	5	-32
2.423	100	20	0	20	0	0	5	1	-34
2.423	100	20	0	20	0	0	5	0.05	-32
2.423	140	0	0	0.2	0	0	10	0.05	-33
2.423	150	0	1.325	3.2	0.337	30	0	5	-36
2.423	100	50	1	1	0	5	20	0.01	-34
2.423	100	30	0	20	0	0	5	0.01	-33
2.423	100	20	1.325	3.2	0.337	30	2.5	0.05	-34
2.423	80	0	0	10	0	0	1	2.1	-36
2.423	150	20	1.325	3.2	0.337	30	2.5	0.002	-33
2.423	150	20	0	0	0.337	30	2.5	5	-32
2.423	100	50	10	30	0.3	55	10	5	-37
2.423	150	0	1.325	3.2	0.337	30	2.5	0	-37
2.423	100	10	10	1	0.3	55	1	0.01	-36
2.423	150	20	1.325	3.2	0	30	2.5	5	-31
2.423	100	30	0	40	0	0	2.5	0.01	-35
2.423	150	20	1.325	3.2	0	0	2.5	5	-30
2.423	100	50	1	30	0.3	55	10	0.01	-38
2.423	100	50	1	1	0.3	5	10	0.01	-32
2.423	100	10	1	1	0.3	5	10	5	-33
2.423	150	0	0	3.2	0.337	30	2.5	5	-37
2.423	100	50	1	1	0.3	5	1	5	-30
2.423	100	30	5.5	30	0.3	30	0	0	-35
2.423	80	0	0	10	0	0	10	0.05	-39
2.423	150	20	1.325	0	0.337	0	2.5	5	-30
2.423	100	50	1	30	0.3	55	1	5	-37
2.423	100	30	1	20	0.337	0	2.5	0	-33
2.423	80	0	0	0.2	0	0	10	0.1	-36
2.423	150	20	0	3.2	0.337	0	2.5	5	-29
2.423	150	20	1.325	0	0.337	30	2.5	0	-32
2.423	100	10	1	1	0.3	0	1	0.01	-31
2.423	100	50	10	1	0.3	5	1	0.01	-32
2.423	150	20	0	3.2	0	30	2.5	5	-33
2.423	80	0	0	0.2	0	0	10	2.05	-36

2.423	150	20	0	3.2	0.337	30	0	5	-32
2.423	150	20	1.325	0	0.337	30	0	5	-33
2.423	150	20	1.325	3.2	0	30	2.5	0	-33
2.423	100	50	10	1	0.3	55	1	5	-34
2.423	100	10	1	1	0.3	55	1	5	-37
2.423	80	0	0	10	0	0	1	0.05	-36
2.423	100	10	1	30	0.3	55	10	5	-41
2.423	80	0	0	0.2	0	0	1	1.05	-33
2.423	100	50	1	1	0.3	55	10	5	-36
2.423	100	50	10	30	0.3	55	1	0.01	-36
2.423	100	50	10	1	0.3	5	10	5	-33
2.423	80	0	0	0.2	0	0	1	2.05	-33
2.423	150	20	1.325	3.2	0.337	0	0	5	-30
2.423	100	10	10	1	0.3	5	1	5	-34
2.423	150	20	1.325	3.2	0.337	0	2.5	0	-31
2.423	100	30	1	20	0.337	0	0	0.01	-33
2.423	80	0	0	0.2	0	0	10	2.05	-37
2.423	100	50	1	1	0.3	55	1	0.01	-35
2.423	150	20	1.325	3.2	0.337	30	0	0	-33
2.423	100	10	1	1	0.3	55	10	0.01	-38
2.423	140	0	0	10	0	0	1	0.05	-35
2.423	100	10	10	1	0.3	55	10	5	-38
2.423	140	0	0	0.2	0	0	1	0.1	-31
2.423	80	0	0	10	0	0	1	0.05	-36
2.423	100	10	1.3	1	0	0	10	0.05	-33
2.423	120	20	1.3	0	0	0	10	0.05	-31
2.423	150	20	1.325	3.2	0.337	30	2.5	2	-28
2.423	100	20	0.5	20	0	0	5	0.05	-34
2.423	100	20	1	20	1	0	5	0.5	-29
